# Pseudouridine guides germline small RNA transport and epigenetic inheritance

**DOI:** 10.1038/s41594-024-01392-6

**Published:** 2024-09-06

**Authors:** Rowan P. Herridge, Jakub Dolata, Valentina Migliori, Cristiane de Santis Alves, Filipe Borges, Andrea J. Schorn, Frédéric van Ex, Ann Lin, Mateusz Bajczyk, Jean-Sebastien Parent, Tommaso Leonardi, Alan Hendrick, Tony Kouzarides, Robert A. Martienssen

**Affiliations:** 1 Howard Hughes Medical Institute, Cold Spring Harbor Laboratory, Cold Spring Harbor, NY USA; 2 Department of Gene Expression, Institute of Molecular Biology and Biotechnology, Adam Mickiewicz University, Poznan, Poland; 3 Gurdon Institute, University of Cambridge, Cambridge, UK; 4 Center for Genomic Science of IIT@SEMM, Instituto Italiano di Tecnologia (IIT), Milan, Italy; 5 Storm Therapeutics, Ltd., Moneta Building (B280), Babraham Research Campus, Cambridge, UK; 6 Present Address: Department of Biochemistry, University of Otago, Dunedin, New Zealand; 7 Present Address: Wellcome Sanger Institute, Wellcome Genome Campus, Hinxton, Cambridge, UK; 8 Present Address: CNRS, INRA Versailles, Versailles, France; 9 Present Address: Inari LLC, Ghent, Belgium; 10 Present Address: Stanford University, Stanford, CA USA; 11 Present Address: Agriculture Canada, Ottawa, Ontario Canada

**Keywords:** Dosage compensation, RNAi

## Abstract

Developmental epigenetic modifications in plants and animals are mostly reset during gamete formation but some are inherited from the germline. Small RNAs guide these epigenetic modifications but how inherited small RNAs are distinguished in plants and animals is unknown. Pseudouridine (Ψ) is the most abundant RNA modification but has not been explored in small RNAs. Here, we develop assays to detect Ψ in short RNA sequences, demonstrating its presence in mouse and *Arabidopsis* microRNAs. Germline small RNAs, namely epigenetically activated small interfering RNAs (easiRNAs) in *Arabidopsis* pollen and Piwi-interacting RNAs in mouse testes, are enriched for Ψ. In pollen, pseudouridylated easiRNAs are transported to sperm cells from the vegetative nucleus, and *PAUSED/HEN5 (PSD)*, the plant homolog of Exportin-t, interacts genetically with Ψ and is required for this transport. We further show that Exportin-t is required for the triploid block: small RNA dosage-dependent seed lethality that is epigenetically inherited from pollen. Thus, Ψ has a conserved role in marking inherited small RNAs in the germline.

## Abstract (web summary)

Germline small RNAs in plants and mammals are marked by pseudouridine. In plants, Exportin-t is required for pseudouridylation, transport and localization of small RNAs in pollen, as well as for epigenetic inheritance in the form the triploid block.

## Main

During gamete formation, epigenetic marks such as histone modification and DNA methylation that accumulate during development are typically reset; however, a subset of these marks, including those marking imprinted genes, are inherited intergenerationally^1^. Unique species of small RNAs are abundant in plant and animal gametes, many of which are cell type specific, and some of which are also inherited by the next generation^2,3^. In the case of *Caenorhabditis* *elegans*, precursors of these intergenerational small RNAs are distinguished by poly(U-G) tails^4^, while the mechanism for small RNA inheritance in plants and in other animals is yet to be elucidated. In the mammalian germline, spermatids accumulate 26–28-nt Piwi-interacting RNAs (piRNAs) that silence transposable elements (TEs), while spermatocytes accumulate 29–30-nt piRNAs that also target genes^5^. In plants, the male germline terminates in mature pollen, which contains two cell types: the pollen grain (or vegetative cell) and two sperm cells enclosed within its cytoplasm. Plants do not have piRNAs; instead, 21–22-nt epigenetically activated small interfering RNAs (easiRNAs) are produced from TEs in the vegetative nucleus (VN) and translocated to the sperm cells^6–8^. Furthermore, 24-nt small interfering RNAs (siRNAs) from some DNA transposons are translocated into developing meiocytes in the anther from the surrounding layer of somatic cells (the tapetum)^7^. Both types of germline small RNA are produced by the plant-specific RNA polymerase, Pol IV (refs. ^7,9,10^) and mediate epigenetic inheritance after fertilization^9,11^. In maize, 21-nt and 24-nt phased secondary siRNAs (phasiRNAs) are also transported from epidermal and tapetal cells into meiocytes, demonstrating that these mechanisms may be conserved^12^. Both microRNAs (miRNAs) and siRNAs in plants are modified by 2′-*O*-methylation, while only piRNAs have this modification in animals^13^.

## Results

### Detecting pseudouridine (Ψ) in miRNAs and their precursors

**Fig. 1:**
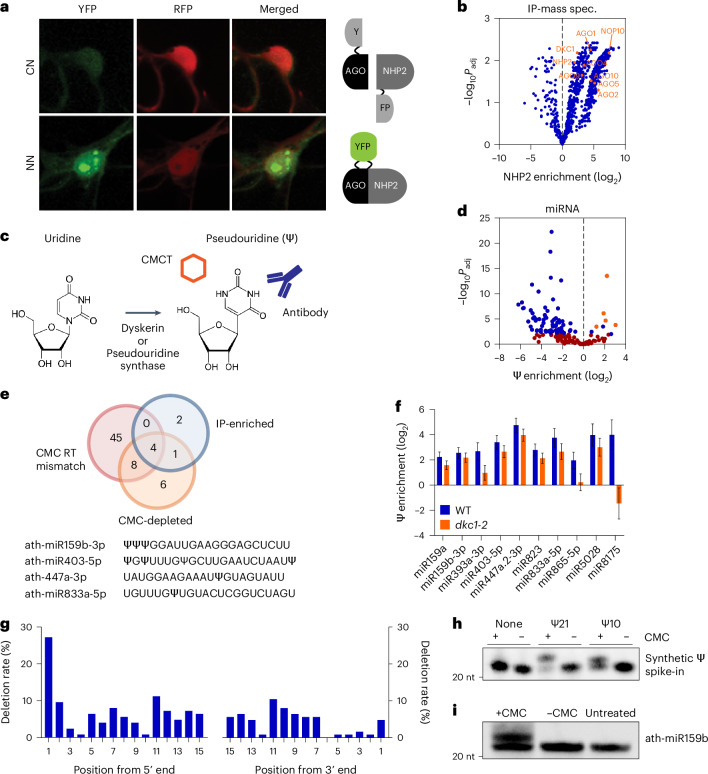
microRNAs are pseudouridylated in plants and mammals. **a**, BiFC confirming interaction of AGO3 with the DSKC1 cofactor NHP2 in *Arabidopsis* (Methods). Split yellow fluorescence protein (YFP) was fused to the C and N termini or both N termini of AGO3 and NHP2. Reconstituted YFP signifies interaction. p35S::RFP (red fluorescence protein) acted as a transformation control. **b**, Volcano plot showing proteins copurified with GFP-tagged NHP2 compared to IP performed in WT plants. NHP and AGO family proteins are depicted in orange (*n* = 3 biological replicates; statistical significance was calculated using edgeR (Methods); *P* value was adjusted by Benjamini–Hochberg correction). **c**, Structure of uridine and Ψ, enzymes involved in catalysis and methods of detection with specific antibodies and chemical modification with CMC (Methods and Extended Data Fig. 2). **d**, Volcano plot of miRNAs enriched by Ψ-IP compared to unbound fractions from *Arabidopsis* flower buds. Blue dots, significantly enriched or unbound miRNAs (adjusted *P* < 0.01); orange, both Ψ-IP enriched and depleted from libraries following CMC treatment (adjusted *P* < 0.01); dark red, not significant. Statistical significance was calculated using DESeq2; P value was adjusted by Benjamini and Hochberg method (*n* = 5 biological replicates). **e**, Venn diagram showing overlap of miRNAs detected in flower buds by each technique. Predicted Ψ sites in miRNAs detected by all three techniques. **f**, miRNAs enriched by Ψ-IP in WT and *dkc1* mutants in *Arabidopsis* leaves. Error bars indicate the s.e.m. of log_2_(fold change) estimated by DESeq2; *n* = 3 WT and n = 4 *dkc1* biological replicates. **g**, Metaplot of modification frequency of miRNAs at each site based on proximity to the 5′ or 3′ end determined by CMC/Mn^2+^ sequencing. **h**, Northern blots of three synthetic 21-mer oligoribonucleotides (sequence: UGACACAGGACUACGGACGUAU) either unpseudouridylated or pseudouridylated at position 10 or 21, treated (+) or mock-treated (−) with CMC and probed with a matching DIG-labeled probe (representative image of three independent experiments). **i**, Flower bud small RNA treated (+), mock-treated (−) or untreated (no mock treatment) with CMC and probed with miR159b to reveal mobility shifts (representative image of four independent experiments). Source data

**Extended Data Fig. 1:**
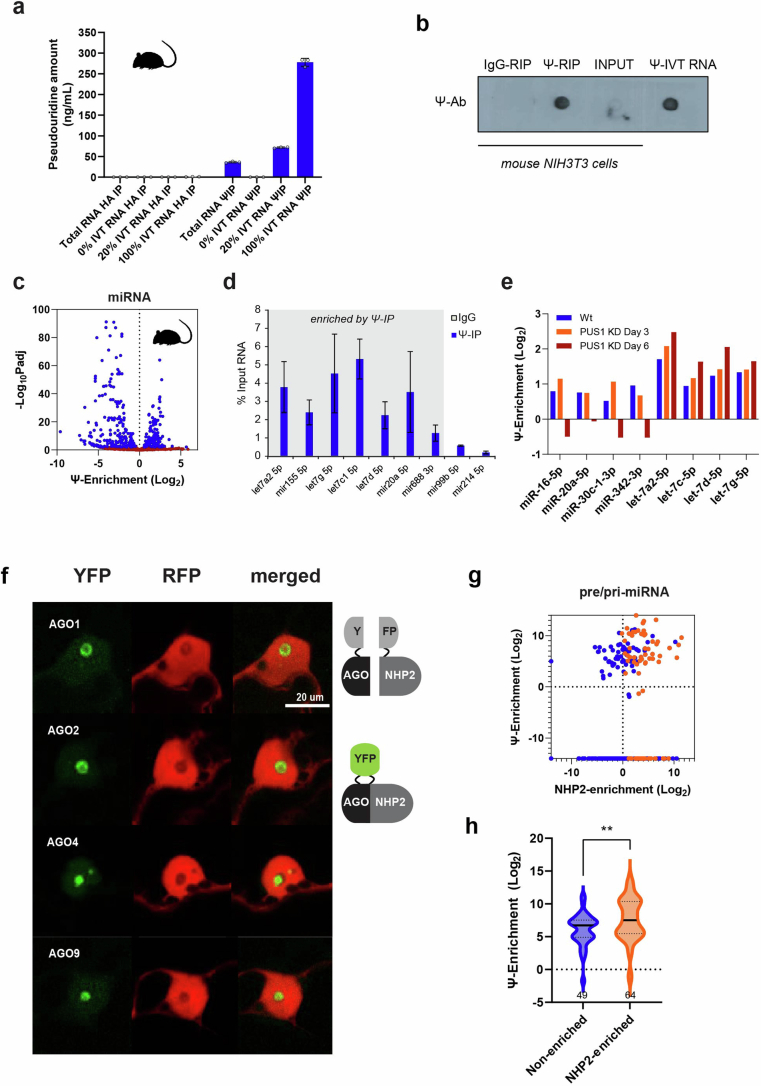
miRNA from plants and mammals are pseudouridylated. (**a**) Immunoprecipitation with anti-Ψ antibody enriches for Ψ-containing RNAs produced through in vitro transcription (IVT) as determined by mass spectrometry (n = 3 biological replicates, error bars ± SD). (**b**) Immuno-dot blot of 100 ng total input RNA or 100 ng RNA immunoprecipitated with anti-Ψ antibody or control immunoglobulin G (IgG). 100 ng of 100% Ψ IVT RNA was used as control. (**c**) Volcano plot of miRNAs enriched by small RNA immunoprecipitation using a Ψ-specific antibody (Ψ-IP) in mouse fibroblast NIH/3T3 cells compared to input fractions. Significantly enriched and depleted miRNAs are highlighted in blue (padj<0.01; DESeq2, Benjamini and Hochberg correction for multiple comparisons; n = 3 biological replicates). (**d**) Validation of enriched miRNAs using qRT-PCR. (**e**) Ψ-enrichment of miRNAs affected by PUS1 knockdown (KD) 3 days and 6 days after PUS1 shRNA transfection of NIH/3T3 cells. (**f**) Arabidopsis AGO proteins and Dyskerin subunit NHP2 were fused at the N-terminus to N- and C- terminally split YFP, respectively. Positive interaction is shown by YFP signal, with 35S::RFP as an expression control. (**g**) Ψ-enrichment of precursor pri-miRNAs in Arabidopsis flower buds plotted against pri-miRNAs bound to NHP2 (blue) and detected by RIP-seq in 2 or more replicates (orange). (**h**) Violin plot showing significant difference between NHP2-enriched and non-enriched pri-miRNA in Ψ enrichment of the mature miRNA by Ψ -IP (Log_2_ IP/IgG; numbers below violins indicate number of miRNA analyzed;one-way ANOVA; **p < 0.01). Source data

We set out to determine whether Ψ was present in small RNAs (Fig. 1). Ψ is the most common noncanonical base in RNA but has not been explored in small RNAs because of difficulties in detection. High-throughput techniques to detect pseudouridylation in longer RNAs specifically label Ψ with *N*-cyclohexyl-*N*′-(2-morpholinoethyl)-carbodiimide-metho-*p*-toluenesulfonate (CMC) to block reverse transcriptase (RTase) during RNA sequencing (RNA-seq) library preparation^14–17^. However, in the case of small RNA, RTase blocks would result in truncated sequences too short to map back to the genome. More recently, anti-Ψ antibody binding^18^ and bisulfite treatment at neutral pH to introduce mismatches^19^ have been used to achieved similar results. In mouse, long primary miRNA precursor transcripts (pri-miRNAs) have been shown to contain Ψ^20,21^; thus, we initially tested for the presence of Ψ in small RNAs from mouse NIH/3T3 cells. Following immunoprecipitation (IP) with a Ψ-specific antibody, we could robustly detect Ψ using a combination of mass spectrometry (MS) (Extended Data Fig. 1a) and dot blots (Extended Data Fig. 1b) using in vitro synthesized pseudouridylated transcripts as a positive control. Using this antibody, we then performed Ψ-IP and small RNA-seq from NIH/3T3 cells before and after knockdown of the gene encoding Ψ synthase, *PUS1* (Extended Data Fig. 1c–e and Supplementary Table 1). We detected Ψ in several miRNAs (Extended Data Fig. 1c), including members of the let-7 family, further validated by qPCR (Extended Data Fig. 1d). However, let-7 was unaffected by *PUS1* knockdown, consistent with its interaction with dyskerin (DKC1) and TruB1 instead^20,21^. Nevertheless, four other pseudouridylated miRNAs were strongly impacted by the *PUS1* knockdown (Extended Data Fig. 1e and Supplementary Table 1).

In search of factors that might modify small RNAs in plants, we performed a yeast two-hybrid screen for interactors of *Arabidopsis* ARGONAUTE 3 (AGO3) using a complementary DNA (cDNA) library of transcripts from *Arabidopsis* flowers^22^. The screen (Methods) identified a potential interactor of AGO3, namely H/ACA RIBONUCLEOPROTEIN COMPLEX SUBUNIT 2/NON-HISTONE PROTEIN2 (NHP2), and we confirmed the interaction by bimolecular fluorescence complementation (BiFC) in phase-separated nucleolar bodies (Fig. 1a). NHP2 has a key role in RNA pseudouridylation by the Ψ synthase DKC1, often though not always through small nucleolar RNA (snoRNA) guides^23–25^. NHP2 was also in close proximity with other AGO proteins, notably AGO4, which has been found in nucleolar bodies and in related Cajal bodies (Extended Data Fig. 1f)^26^. IP of green fluorescent protein (GFP)-tagged NHP2 followed by MS revealed interactions with other subunits of the DKC1 complex (NOP10), as well as components of the RNA interference machinery, including AGO1, AGO2, AGO4, AGO5, AGO9 and AGO10 (Fig. 1b and Supplementary Table 2). IP of GFP-tagged NHP2 followed by RNA-seq revealed snoRNAs, small nuclear RNAs (snRNAs) and several pri-miRNAs that coimmunoprecipitated with NHP2 either directly or as part of a larger complex (Supplementary Table 3 and Extended Data Fig. 1g). To determine whether pri-miRNAs are pseudouridylated in plants, as they are in animals, we then performed Ψ-IP and sequencing on mRNAs from *Arabidopsis* flower buds (collectively called the inflorescence). Several pri-miRNA transcripts were enriched and there was a tendency for those enriched by IP with GFP-tagged NHP2 to also be enriched by Ψ-IP (*P* < 0.01, determined by analysis of variance (ANOVA)) (Extended Data Fig. 1h and Supplementary Table 4).

### Mapping Ψ in miRNAs

**Extended Data Fig. 2:**
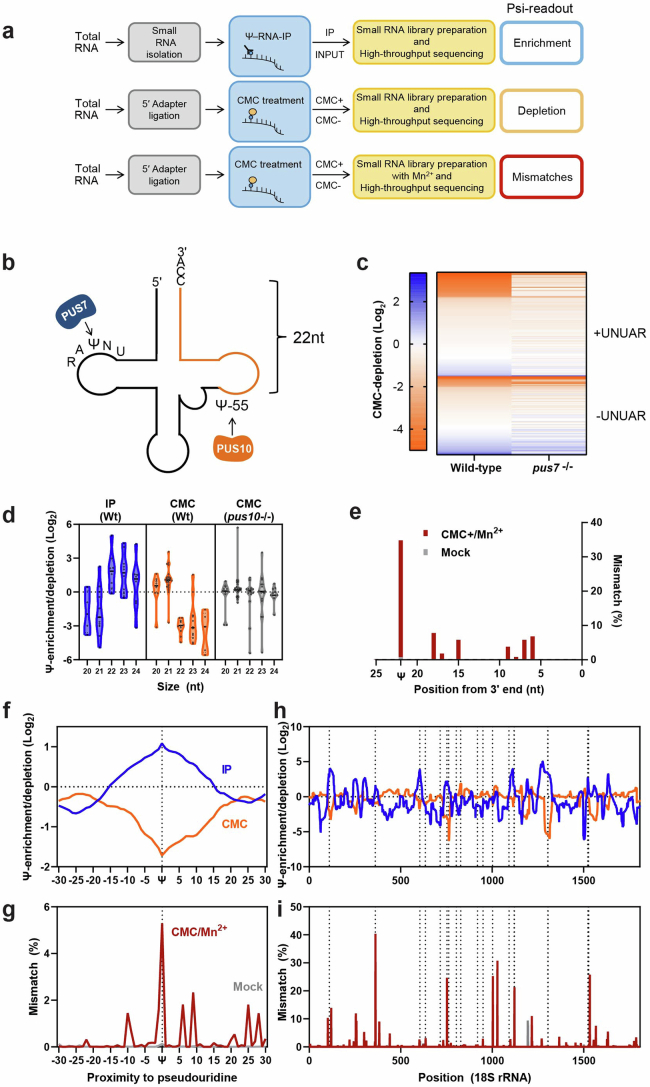
Strategies for robust detection of Ψs in small RNAs. (**a**) Three strategies to detect Ψ in small RNAs and their predicted effect on pseudouridylated sequences. Details are in Methods (**b**) Known sites of pseudouridylation in tRNAs mediated by PUS7 (targets UNUAR) and PUS10 (targets Ψ-55). Ψ-55 is 22 nt ‘upstream’ of the 3ʹ end of -CCA tailed tRNA fragments. (**c**) Heatmap showing individual tRNA fragments (tRFs) with and without the UNUAR motif in wild-type and *pus7*−/− libraries treated with CMC. (**d**) 3′CCA tRFs ≥22 nt contain canonical Ψ-55 and are enriched by anti-Ψ antibody and depleted by CMC treatment, whereas shorter fragments do not contain this nucleoside and are not enriched/depleted. Ψ-55 is ablated in mutants of PUS10. (**e**) Deletions in 3ʹ CCA tRFs based on position in CMC/Mn^2+^- and mock-treated libraries. (**f, g)** Metaplots showing coverage depletion/enrichment in CMC/IP libraries at rRNA pseudouridylation sites (**f**) compared to mismatches from Mn2 + /CMC+ libraries (**g**). Average across 42 predicted pseudouridylated sites in 18S and 25S rRNA and from 17 predicted sites in 18S rRNA (CMC mismatch/Mock mismatch). (**h, i**) Enrichment/depletion/mismatches across 18S rRNA in CMC/IP (**h**) and CMC/Mn^2+^ (**i**) libraries. Predicted Ψs are marked with dotted lines. Source data

Encouraged by these results, we tested three approaches to detect Ψ directly in small RNAs in *Arabidopsis* (Methods, Fig. 1c and Extended Data Fig. 2a): (1) Ψ-IP followed by small RNA-seq; (2) CMC treatment of small RNA libraries following 5ʹ-adapter ligation to block RTase and deplete small RNAs that contain Ψ; and (iii) reverse transcription (RT) of CMC-treated small RNAs in the presence of Mn^2+^, which causes deletions and mutations at Ψ residues instead of an RT stop^27^. To determine the efficacy of these techniques, we examined coverage at known pseudouridylation sites in ribosomal RNA (rRNA) and transfer RNA (tRNA). Ψ at UNΨAR motifs is directed by PUS7 and was detected by CMC depletion in the wild type (WT) but not in *pus7* mutants (Extended Data Fig. 2b,c). Ψ at position 55 in tRNA depends on PUS10 (Extended Data Fig. 2b). tRNA fragments containing Ψ-55 were IP enriched and CMC depleted, contained mismatches in CMC/Mn^2+^-treated libraries and were ablated in *pus10* mutants (Extended Data Fig. 2b,d,e). Predicted sites of rRNA pseudouridylation were also enriched by Ψ-IP, depleted by CMC treatment and mismatched in CMC/Mn^2+^ libraries (Extended Data Fig. 2f–i). Thus, all three methods robustly detected Ψ in small RNA fragments derived from tRNA and rRNA.

**Extended Data Fig. 3:**
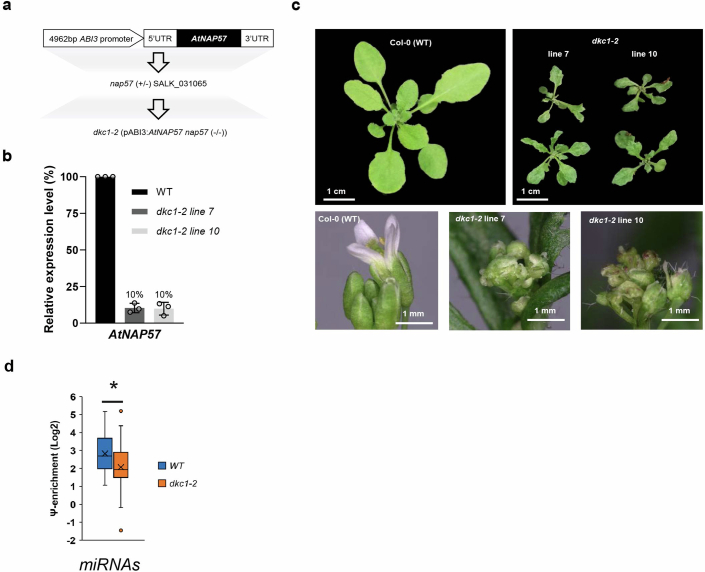
Arabidopsis mutants in Dyskerin/NAP57. **(a**) Generation of *dkc1-2* transgenic plants*. nap57/dkc1* (SALK_031065 T-DNA insertion) was complemented with the pABI3:AtNAP57 transgene (**b**) AtNAP57 transcript level in *dkc1-2* transgenic lines analyzed by qPCR (n = 3 biological replicates; error bars show mean and SD) (**c**) *dkc1-2* transgenic plants show severe developmental defects including dwarfism, abnormal leaf shape, deformed and infertile flowers. (**d**) Enrichment of miRNAs by Ψ-IP in wild-type and *dkc1-3* leaves based on DESeq2 analysis. Boxes show median, upper/lower quartiles and the Tukey min/max with outliers represented as dots, X marks the mean value. N = 3 biological replicates, 44 miRNAs with L2FC > 1, p-adj<0.01 are plotted; significance calculated using Mann-Whitney test, p = 0.003. Source data

We then applied our assays to detect Ψ in mature small RNAs from *Arabidopsis* inflorescence and detected more than 50 pseudouridylated mature miRNAs (Fig. 1g), of which four (miR159b-3p, miR403-5p, miR447a-3p and miR833a-5p) were detected using all three assays (Fig. 1d,e and Supplementary Tables 5–7). The *pus7* and *pus10* mutants had no detectable effect on Ψ-IP enrichment or CMC depletion of miRNA but we expected that DKC1 might be responsible, given the enrichment for pri-miRNA in pulldowns with the DKC1 cofactor NHP2 (Extended Data Fig. 1b). As mutants of NHP2 and other members of the DKC complex are lethal^28^, we rescued *dkc1-*knockout mutations with an embryo-specific DKC1 cassette (Extended Data Fig. 3a), which restored about 10% of WT *DKC1* expression in the leaves (Extended Data Fig. 3b) and allowed seeds to germinate into viable but infertile plants (Extended Data Fig. 3c). Several miRNAs were significantly enriched by Ψ-IP in WT leaves (Fig. 1f), of which 3/10 required DKC1 for pseudouridylation (Fig. 1i). We conclude that, just as some miRNAs are pseudouridylated by PUS1 in the mouse (Extended Data Fig. 1h), some are pseudouridylated by DKC1 in *Arabidopsis*; however, in both cases, multiple Ψ synthases must be responsible for miRNA pseudouridylation, just as they are for mRNA pseudouridylation in plants, yeast and mammals^29^.

**Extended Data Fig. 4:**
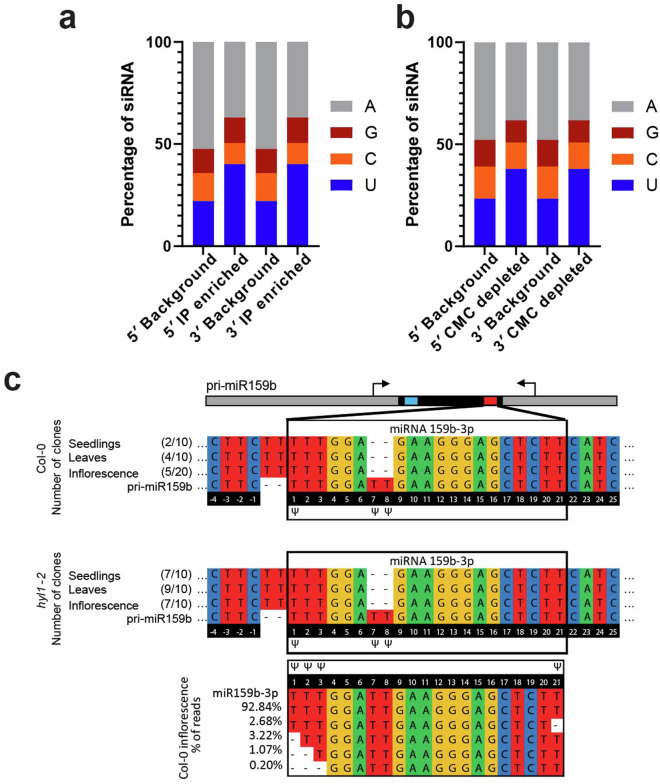
Mapping of miRNA pseudouridylation sites in Arabidopsis by CMC labeling and reverse transcription in the presence of Mn^2+^ (CMC/Mn^2+^). (**a**, **b)** Bar chart showing proportions of 5ʹ and 3ʹ nucleotides in Ψ-IP-enriched (**a**) and CMC-depleted (**b**) siRNAs from inflorescence compared to background (non-structural) small RNAs. IP-enrichment and CMC-depletion show biases towards 5ʹ and 3ʹ U. (**c**) Sites of predicted Ψ (insertions and deletions) in precursor pri-miR159b PCR amplicons (upper panels) and mature miR159b (lower panel; high-throughput sequencing) in seedlings, leaves and flower buds of wild-type and *hyl1-2* microprocessor mutants. pri-miRNA amplicons (upper) and mature small RNA sequences (lower) had insertions and deletions at predicted sites. Number of clones with the respective amplicon is shown in brackets. Source data

Ψ detection by mismatch sequencing revealed that pseudouridylation in *Arabidopsis* miRNAs (Fig. 1g) was prevalent at the 5ʹ nucleotide, which is frequently uridine in miRNAs and contributes to AGO loading preference^30–32^. Similarly, siRNAs detected by Ψ-IP enrichment and CMC depletion were enriched for 5′ and 3′ U (Extended Data Fig. 4a,b). As pri-miRNAs associate with NHP2 and contain Ψ (Extended Data Fig. 1g), we predicted that Ψ in mature miRNAs was deposited before processing of pri-miRNAs into small RNAs by the endonuclease Dicer. cDNA reverse-transcribed from pri-miR159b in the presence of CMC and Mn^2+^ revealed extensive mismatches (pseudouridylation) at the 5ʹ dicing site (25% Ψ), which was exacerbated in a *hyponastic leaves 1 (hyl1)* mutant (75% Ψ) that accumulates pri-miRNAs because of loss of the microprocessor component R2D2 (Extended Data Fig. 4c). Ψ was also detected at 5ʹ and 3ʹ sites in mature miR159b, suggesting that pseudouridylated pri-miRNAs are processed into mature miRNAs (Extended Data Fig. 4c). We confirmed the presence of Ψ in mature miR159b on northern blots, where CMC treatment induces a mobility shift in pseudouridylated small RNAs (Fig. 1h,i). Intriguingly, miR159b is inherited from sperm cells by the fertilized endosperm, where it impacts seed development^3^. As miR159b is heavily pseudouridylated (Fig. 1i), this suggests that pseudouridylated small RNA may be inherited from pollen.

### Ψ is strongly enriched in germline small RNAs

**Fig. 2:**
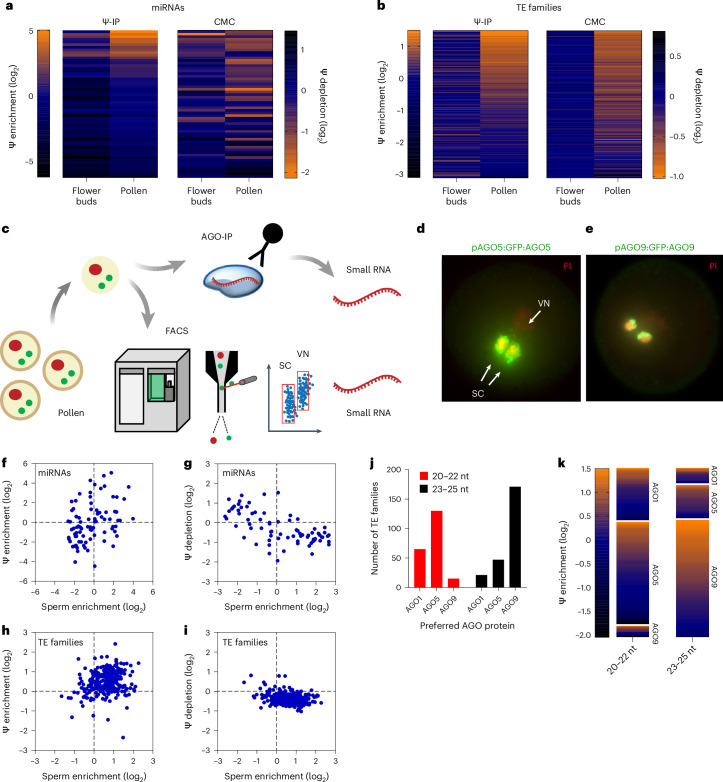
Pollen siRNAs are highly pseudouridylated and loaded into AGO proteins in sperm cells. **a**, Heat maps of individual miRNA Ψ enrichment in flower buds and pollen detected by Ψ-IP (log_2_ IP/unbound; *n* = 5 biological replicates) and by CMC-mediated depletion from small RNA libraries (log_2_ CMC^+^/CMC^−^; *n* = 2 treatment replicates) and assessed by DESeq2. **b**, Heat maps of siRNA Ψ enrichment from individual TE families in flower buds and pollen detected by Ψ-IP and CMC depletion and assessed as in **a**. Small RNAs from most TE families were enriched for Ψ in pollen. **c**, Small RNAs were sequenced from sperm cells isolated from pollen grains by FACS, followed by RIP-seq with antibodies to AGO proteins. SC, sperm cell. **d**, GFP::AGO5 fusion protein expressed from *AGO5* promoter (*pAGO5*) in mature pollen is found in the perinuclear space around sperm cells. DAPI was used to stain the VN. Reproduced with permission from Borges et al.^34^. **e**, AGO9::GFP fusion protein expressed from the *AGO9* promoter (*pAGO9*) in mature pollen. **f**–**i**, Association of small RNA pseudouridylation in pollen, determined by Ψ-IP (**f**,**h**) and CMC depletion (**g**,**i**) with sperm cell abundance by FACS, for both miRNAs (**f**,**g**) and siRNAs from TE families (**h**,**i**)^6,34^. **j**, Bar chart showing number of preferred TE families for each AGO protein split by siRNA size (20–22 and 23–25 nt). Each TE family was assessed for enrichment in AGO1, AGO5 and AGO9 IP as a function of two size classes (20–22 and 23–25 nt), with the highest enrichment signaling a preference for a certain AGO. **k**, Heat maps showing siRNA Ψ enrichment from individual TE families split by AGO preference and size class. Source data

**Extended Data Fig. 5:**
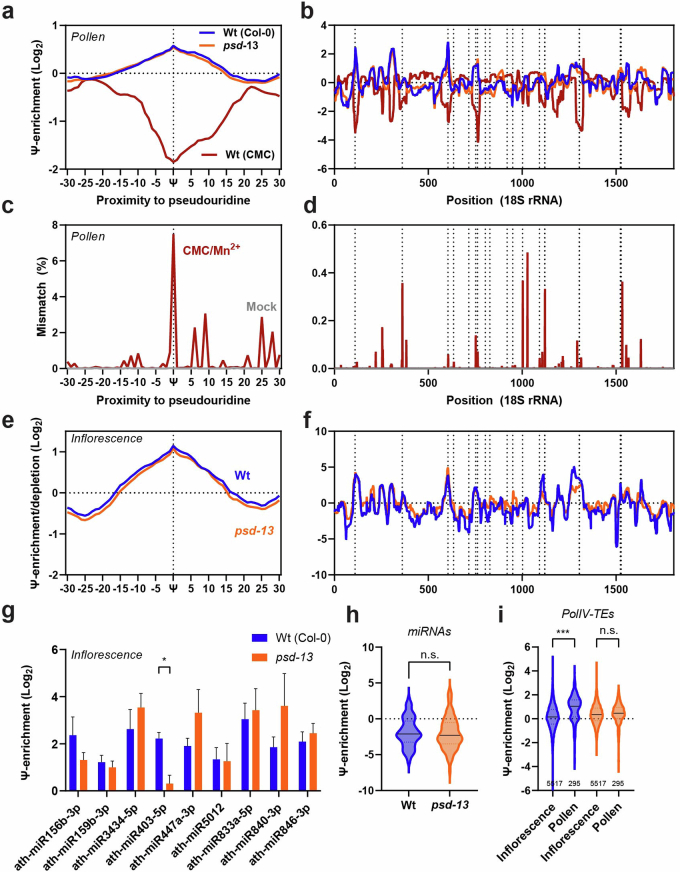
Pseudouridine in miRNAs and TE-derived siRNAs in pollen from WT and *paused (psd-13)/*exportin-t mutants. (**a**) Metaplots of read coverage at 42 known pseudouridylated sites in 18S and 25S rRNA, relative to untreated controls, in CMC-treated WT (magenta) and Ψ-IP WT (blue) and *psd-13* (orange) small RNA sequencing libraries from pollen. (**b**) Read coverage from the same libraries at 17 rRNA pseudouridylation sites in 18S rRNA. (**c, d**) metaplot (**c**) and browser (**d**) analysis of predicted sites in rRNA from CMC/Mn^2+^ analysis relative to mock treated controls. (**e, f**) Metaplots (**e**) and read coverage (**f**) relative to untreated controls in WT (blue) and *psd-13* (orange) Ψ-IP small RNA sequencing libraries from inflorescence. Known positions of Ψ in **b**, **d** and **f** are marked with dotted lines. (**g**) Ψ-enrichment of individual miRNAs detected by Ψ-IP and small RNA sequencing in WT and *psd* mutant inflorescence (L2FC calculated by DESeq2 with estimate of SE; n = 5 and 2 biological replicates for WT and *psd*, respectively; miR403 p = 0.01027, two-tailed students t-test). (**h**) Ψ-enrichment of miRNAs overall in WT and *psd* mutant inflorescence (two-way ANOVA, p = 0.2747; n = 153 miRNAs) (**i**) Violin plot of Ψ-enrichment of siRNAs from individual TEs based on IP-enrichment (Log_2_ IP/Unbound) in flower buds (inflorescence) and pollen from wild-type and *psd* mutants. Significant Ψ enrichment was observed in pollen, but not in inflorescence, and depended on *PSD* (p < 0.0001; p = 0.9181, one-way ANOVA; numbers under violins indicate number of TEs analyzed). Asterisks in g and i indicate significance (p < 0.05 (*); p < 0.01 (**); p < 0.001 (***)). Source data

Therefore, we applied Ψ-IP, CMC and CMC/Mn^2+^ treatments to small RNA isolated from pollen. We found that known sites of pseudouridylation in 18S rRNA were robustly detected in rRNA fragments in pollen by all three techniques (Extended Data Fig. 5a–d). In both CMC and Ψ-IP datasets, miRNAs in pollen had a much greater level of pseudouridylation than in flower buds (Fig. 2a and Supplementary Tables 7–9). This effect was even more dramatic for siRNAs, which were heavily pseudouridylated in pollen (Fig. 2b). Pseudouridylated siRNAs (Ψ-siRNAs) were associated with most annotated TE families, although there were notable exceptions (for example, AtGP2N and AtGP2). Next, we tested whether Ψ-miRNAs and Ψ-siRNAs were enriched in sperm cells purified by fluorescence-activated cell sorting (FACS) or were immunoprecipitated with AGO proteins from total pollen (Fig. 2c). AGO2, AGO5, AGO6, AGO7 and AGO9 bind siRNA and are restricted to sperm cells in mature pollen^33^, where AGO5 and AGO9 are localized to the perinuclear space (Fig. 1d,e). AGO1 on the other hand binds mostly miRNA and accumulates in both sperm cells and the VN^33^.

To determine whether pseudouridylation of small RNA reflects their localization, we compared the pseudouridylation of pollen miRNAs to sperm cell localization. As expected from previous studies^34^ and consistent with AGO1 localization, miRNAs were found in both sperm cells and pollen and some miRNAs in each case were pseudouridylated (Fig. 2f,g). Consistent with the localization of AGO2, AGO5, AGO6, AGO7 and AGO9, however, siRNAs were strongly enriched in sperm cells and siRNAs from most TE families were pseudouridylated (Fig. 2h,i). To determine whether individual AGO proteins preferentially bound pseudouridylated siRNAs, we performed small RNA-seq after IP (RIP-seq) with anti-AGO9 antibodies (Methods) and compared the abundance of siRNAs of each size class in each TE family to published RIP-seq data from AGO1 and AGO5 (ref. ^35^), with AGO5 and AGO9 being preferred for the size classes of 20–22 nt and 23–25 nt, respectively (Fig. 2j). Next, we examined pseudouridylation levels of these families by RIP-seq to determine whether AGO binding influenced Ψ levels and we found that all AGO proteins bound pseudouridylated siRNA from a similar proportion of TE families (Fig. 2k). This was consistent with association of the pseudouridylation machinery with all of these AGO proteins by IP–MS (Fig. 1b). We, therefore, sought to determine what other factors might control pseudouridylation in pollen.

### Germline localization and pseudouridylation depend on Exportin-t

Pseudouridylation has been implicated in the cytoplasmic localization of mRNA and tRNA, suggesting a role for Ψ in small RNA transport^16,36^. Pol IV-dependent 21–22-nt easiRNAs in pollen are also mobile and move into sperm cells from the VN^6–8^. To further investigate a role for Ψ in intercellular mobility of small RNAs, we probed the function of Ψ in sperm cell localization. Because our *dkc1* mutant was completely sterile (Extended Data Fig. 3c), we could not use this to investigate the function of Ψ in pollen. Additionally, other PUS enzymes likely have considerable redundancy, as 20 exist in *Arabidopsis*^29^. Pseudouridylation of tRNAs by Pus1^+^ contributes to nuclear export in yeast and genetically interacts with Loss of Suppression 1 (Los1^+^), which encodes the tRNA export factor Exportin-t^36^. Exportin-t binds the upper minihelix of L-shaped tRNA, consisting of the acceptor stem and the TψC loop and terminating at Ψ55 and C56 (ref. ^37^). This minihelix includes the 22-nt 3′ tRNA fragment with a 5′ Ψ and 3′ CCA, resembling a 21–22-nt small RNA duplex^38^. Intriguingly, the *Arabidopsis* homolog of *Los1*^+^, which is known as *PAUSED/HUA ENHANCER5 (PSD/HEN5)*, was previously identified in genetic screens for small RNA phenotypes, along with mutants in *HEN1 (2’-O-methyltransferase)*, *ARGONAUTE10 (pinhead)*, *ARGONAUTE7* (*zippy*), and *HASTY (hst*). *HST* encodes Exportin-5 and was recently shown to be required for long-range transport of artificial miRNA in *Arabidopsis*^39–42^. We, therefore, investigated whether *psd* mutants could be used as a proxy for the function of Ψ in siRNA, as mutants are viable and fertile.

**Fig. 3:**
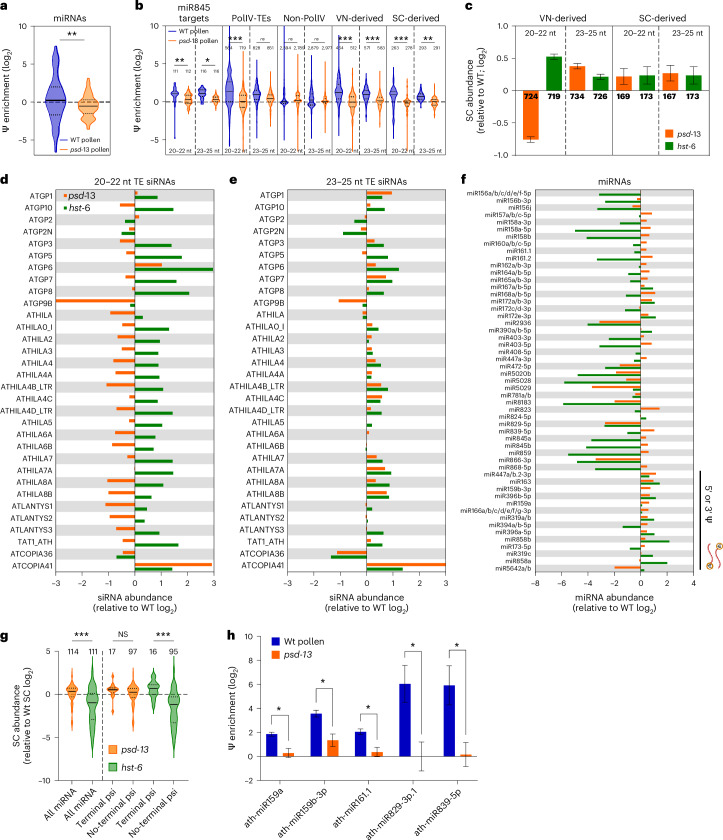
Transport of pseudouridylated siRNAs into sperm cells depends on *PAUSED* (Exportin-t). **a**, Ψ enrichment of miRNAs in WT and *psd*-13 pollen. A modest reduction in Ψ enrichment of miRNAs was observed in *psd* pollen (*P* = 0.0019, determined by two-tailed paired *t*-test; *n* = 3 biological replicates; 64 miRNAs are plotted). **b**, Ψ enrichment of siRNAs was determined by Ψ-IP and small RNA-seq in WT and *psd-13* mutant pollen and plotted by size class (20–22 nt or 23–25 nt) for individual TEs belonging to each group, namely, miR845 targets, Pol IV and non-Pol IV targets and TEs whose siRNAs were derived from sperm cells (SC) or from the VN in ref. ^44^. *P* values show differences between group means, determined by one-way ANOVA with Bonferroni correction for multiple comparisons (*P* = 0.002, 0.015, <0.0001, 0.234, 0.5964, 0.5153, <0.0001, 0.0009, 0.0002 and 0.0049; *n* = 3 biological replicates). Numbers above violins indicate the number of individual TEs plotted (including replicates). **c**, siRNA abundance in SC was determined by sequencing small RNA from FACS-sorted WT, *psd*-13 and *hst*-6 SC. VN-derived 20–22-nt siRNAs were depleted from *psd* SC but enriched in *hst* SC relative to WT, while VN-derived 23–25-nt siRNAs and SC-derived siRNAs were at levels similar to WT. Numbers on bars indicate the number of individual TEs plotted (including replicates); error bars indicate the s.e.m. **d**,**e**, Abundance of highly prevalent siRNA from 30 Gypsy and 2 Copia TE families in *psd* and *hst* sperm relative to WT for 20–22 nt (**d**) and 23–25 nt (**e**) size classes. **f**, Abundance of highly prevalent miRNAs (reads per million mapped reads (RPM) > 100) in *psd* and *hst* sperm relative to WT. miRNAs with terminal Ψ are indicated with a black bar; miRNAs presented in **h** are shown in bold. **g**, Abundance of miRNAs with and without terminal Ψ in *psd* and *hst* mutants relative to WT in sperm cells sorted by FACS. miRNAs without terminal Ψ were lost in *hst*, but not in *psd* sperm cells (*P* < 0.0001, 0.6571 and <0.0001, determined by two-way ANOVA; *n* = 3 biological replicates). Numbers above violins indicate the number of individual miRNAs plotted. **h**, Ψ enrichment of miRNAs in WT and *psd-13* pollen for those miRNAs dependent on *PSD* for pseudouridylation (*P* = 0.02, 0.02, 0.021, 0.037 and 0.039, determined by two-sided Student’s *t*-test; *n* = 3 biological replicates). Error bars indicate the estimated s.e.m. of log_2_(fold change) calculated by DESeq2. In **a**,**b**,**g**,**h**, *P < 0.05, **P < 0.01 and ***P < 0.001. Source data

**Extended Data Fig. 6:**
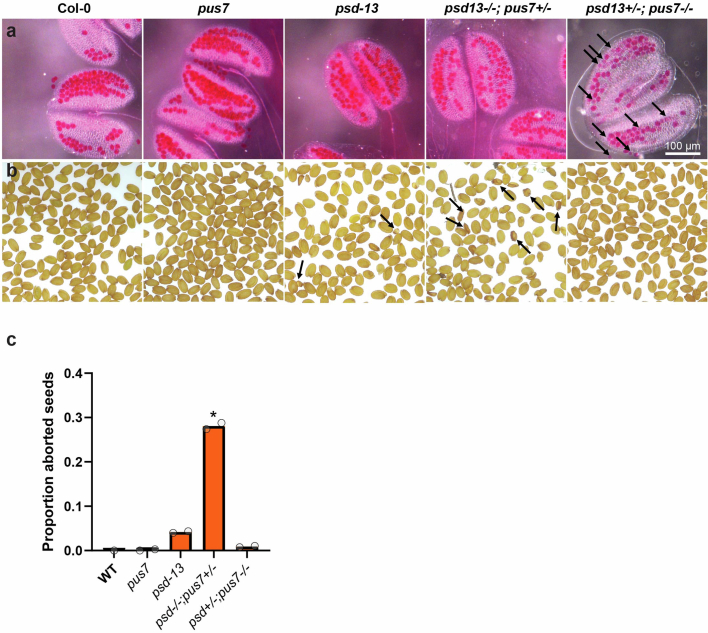
Mutation of *psd* and *pus7* is synthetically lethal in Arabidopsis. (**a**) Alexander staining of pollen from Col-0, *pus7*, *psd-13*, *psd-13*−/−; *pus7* + /−, and *psd-13* + /−; *pus7*−/− parents. *psd*-13 + /; *pus7*−/− had semisterile pollen abortion (loss of Alexander staining, arrows). (**b**) Mature seeds from each parent. Aborted seeds in *psd-13*are highlighted with arrows. (**c**) Quantification of approximately 500 seeds from each genotype revealed *psd*-13−/−; *pus7* + /− aborts seeds at a rate of ~25% (* indicates no difference from expected 3:1 normal:aborted ratio, p > 0.1 Chi-square test). Double homozygous mutants could not be obtained (n = 150 plants). Source data

**Extended Data Fig. 7:**
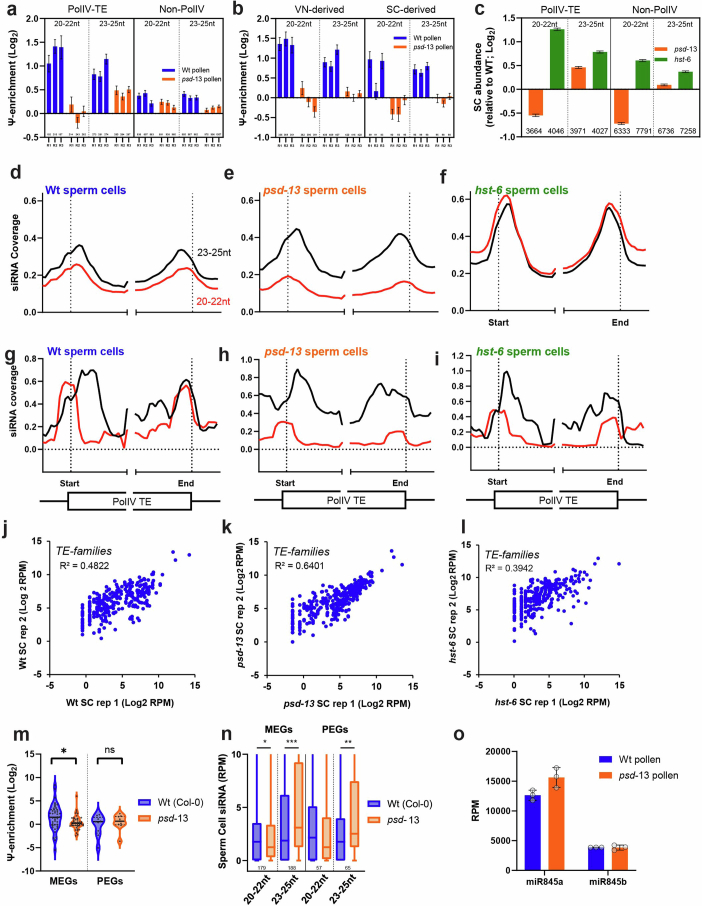
Characterization of pseudouridylation of siRNA in *psd-13* mutants. (**a,b**) Ψ-enrichment of PolIV-dependent and -independent (**a**) or VN- and SC-derived (**b**) TE-siRNAs in wild-type and *psd*-13 pollen for each size class (average enrichment of TE-siRNAs from each classification is shown +/− s.e.m. for each biological replicate; numbers below bars indicate number of TEs analyzed). (**c**) siRNA abundance was determined by sequencing small RNA from FACS sorted wild-type, *psd*-13 and *hst*-6 sperm cells. SC abundance of PolIV-dependent and non-PolIV-dependent TE-siRNAs was assessed in each size class (average enrichment of TE-siRNAs relative to wild-type from each TE classification is shown +/− s.e.m.; numbers below bars indicate number of TEs analyzed). (**d-i**) Metaplots of 20-22nt (red) and 23-25nt (black) siRNA abundance across PolIV-TEs from biological replicates of wild-type (**a**,**d**), *psd-13* (**b**,**e**) and *hst-6* (**c**,**f**) sperm cells sorted by FACS. 250 bp up- and down-stream regions as well as 500 bp from each end of the TE are shown. (**j-l**) Scatterplots of small RNA sequencing data from two biological replicates mapped to TE families in wild-type (**j**), *psd-13* (**k**) and *hst-6* (**l**) sperm cells isolated by FACS. Simple linear regression used to determine R-squared value. (**m**) Ψ-enrichment of siRNAs from TEs surrounding maternally expressed imprinted genes (MEGs, n = 42) and paternally expressed imprinted genes (PEGs, n = 9) in wild type (blue) and *psd-13* (orange) pollen. * = p < 0.05, two-sided paired t-test. (**n**) Abundance of 20-22 nt and 23-25 nt siRNA surrounding imprinted genes in wild-type (blue) and *psd-13* (orange) sperm cells. siRNA abundance in sperm cells matching MEGs and PEGs was lower in *psd-13* mutants for 20-22nt siRNA but relatively higher for 23-25nt siRNA. * = p < 0.05; ** p < 0.01, *** = p < 0.001, paired t-test with Bonferroni correction for multiple comparisons; numbers below boxes indicate individual TEs analyzed. Boxes indicate median, interquartile range and min/max (y-axis clipped to visualize median). (**o**) Levels of miR845a and miR845b were unchanged or higher in *psd-13* mutant pollen (Bars show average RPM; error bars show SD of biological replicates, n = 3). Source data

First, we demonstrated that the *psd* mutation in *Arabidopsis* is synthetically lethal with *pus7* in a manner similar to *los1*^−^ and *pus1*^−^ from yeast. We found that *pus7*^*−/−*^;*psd*^+/−^ double mutants were semisterile (Extended Data Fig. 6a). In contrast, *pus7*^+/−^*;psd*^*−/−*^ plants had fertile pollen but had 25% aborted seed compared to less than 5% in *psd* alone (Extended Data Fig. 6b,c). Viable double homozygous seeds were not recovered from either genotype upon self-fertilization. These results indicated that tRNAs require *PUS7* for modification in diploid cells before meiosis (*pus7*^*−/−*^) and subsequently require *PSD* for nuclear export in haploid pollen (from *psd*^+/−^ parents). Next, we performed Ψ-IP and CMC depletion on small RNA from WT and *psd* mutant pollen, as well as WT and mutant inflorescence (flower buds), and found robust detection of pseudouridylation sites in rRNA (Extended Data Fig. 5a–f). We sought to determine whether *psd* had an effect on pseudouridylation of miRNAs and siRNAs in inflorescence and pollen. Of those miRNAs that were significantly pseudouridylated in the inflorescence, only miR403 lost Ψ in the mutant (Extended Data Fig. 5g), while miRNAs overall were unaffected (Extended Data Fig. 5h). Similarly, siRNAs from TEs were mostly unmodified in the inflorescence and were unaffected by *psd* (Extended Data Fig. 5i). In pollen, however, miRNAs were modestly depleted of Ψ in *psd* relative to WT (Fig. 3a; *P* < 0.01, determined by ANOVA) while easiRNAs were strongly depleted of Ψ (Fig. 3b). easiRNA biogenesis requires both Pol IV and the most abundant miRNAs in pollen, miR845a and miR845b, which target long terminal repeat (LTR) retrotransposons at their RT primer binding site^9,35^. The 20–22-nt easiRNAs that depend on miR845b were heavily pseudouridylated and this depended on *psd*, as did pseudouridylation of 20–22-nt easiRNAs and 23–25-nt siRNAs that depend on Pol IV (Fig. 3b and Extended Data Fig. 7a). However, Pol IV-independent siRNAs had very little Ψ, which was unaffected in *psd* (Fig. 3b and Extended Data Fig. 7a).

In pollen, Pol IV is prominently expressed in the VN^43^, suggesting that this is where pseudouridylation likely takes place. However, easiRNAs accumulate in sperm cells and are thought to be transported there from the VN^6–8^. In a recent study, small RNA biogenesis in the VN and in the sperm cells were independently suppressed by expressing the RNase III-like protein RTL1 in each cell type^44^. We found that both VN-derived and sperm-cell-derived small RNAs were enriched for Ψ and that enrichment depended on *psd* (Fig. 3b and Extended Data Fig. 7b). To determine whether PSD affected siRNA transport from the VN into sperm cells, we isolated sperm cells from mutant *psd-13* (Exportin-t), mutant *hst-6* (Exportin-5) and WT pollen by FACS and compared the abundance of siRNAs and miRNAs (Fig. 3c–e and Extended Data Fig. 7c–l). Strikingly, 21–22-nt easiRNAs were reduced in *psd* sperm cells and elevated in *hst* sperm cells, but only those that were derived from the VN and not for those derived from the sperm cells (Fig. 3c,d). In contrast, 23–24-nt siRNA abundance in sperm cells was unaffected by the *psd* and *hst* mutants (Fig. 3c,e). However, both Pol IV and non-Pol IV TE siRNAs were reduced in *psd* sperm cells, suggesting that Ψ is not strictly required for transport (Extended Data Fig. 7c).

HST (Exportin-5) is known to be required for the export and accumulation of miRNAs^45^ and miRNA levels in sperm cells were significantly reduced in *hst* mutants (Fig. 3f,g). As expected, most miRNAs in sperm cells were unaffected by *psd* (Fig. 3f,g) but individual miRNAs including miR159 and miR403 significantly lost Ψ (Fig. 3h). A likely explanation is that miRNAs that lose Ψ in *psd* mutants can use the *HST* pathway instead to accumulate in sperm cells. One prediction of this model is that Ψ-miRNAs might be able to use the PSD pathway in *hst* mutants. This prediction was confirmed as miRNAs without 5′ terminal Ψ were lost in sperm cells from *hst* mutants, while those with terminal Ψ were retained, suggesting that PSD was sufficient for their transport (Fig. 3f,g and Supplementary Table 7). Thus, *PAUSED/EXPORTIN-T* is required for the transport of pseudouridylated easiRNAs into sperm cells from the VN, while *HASTY/EXPORTIN-5* transports unmodified miRNAs. This novel function for *PAUSED/EXPORTIN-T* is pollen specific, as are most pseudouridylated miRNAs and easiRNAs, which are unaffected by *psd* in the inflorescence (Extended Data Fig. 5g–i). It is noteworthy that tRNAs were found to carry a strong signal for intercellular transport in plants, such that mRNA–tRNA fusions are mobile^46^. It is tempting to speculate that Ψ and *PSD* may be involved in the intercellular transport of tRNA fusions.

### Exportin-t impacts siRNAs at imprinted genes to mediate the triploid block

**Fig. 4:**
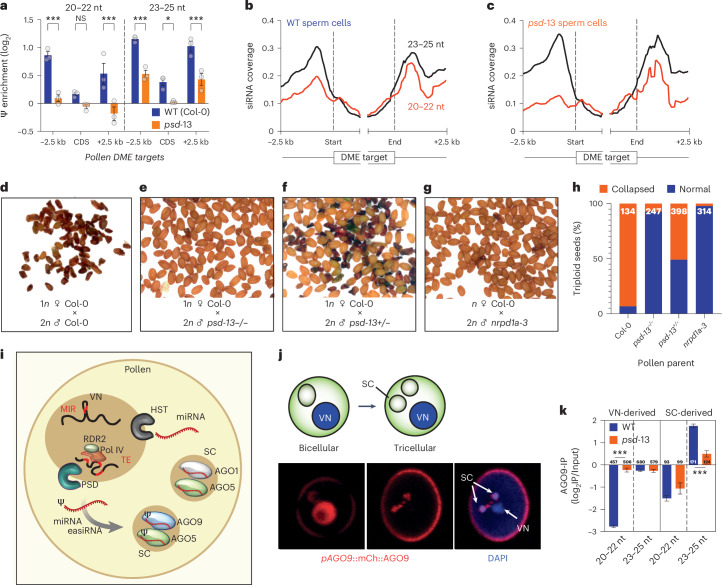
Ψ and Exportin-t mediate epigenetic inheritance and the triploid block. **a**, Mean Ψ enrichment (log_2_ Ψ-IP/unbound) of 20–22-nt siRNAs and 23–25-nt siRNAs surrounding imprinted genes (−2.5 kb upstream of start; CDS; +2.5 kb downstream of stop) targeted by *DME* in WT (blue) and *psd-13* (orange) pollen (*P* = <0.0001, 0.4528, <0.0001, 0.0002, 0.0428 and 0.0003, determined by two-way ANOVA with Bonferroni correction for multiple comparisons; *n* = 3 biological replicates). Error bars indicate the s.e.m. CDS, coding sequence. **b**,**c**, Metaplots of sperm cell abundance of 20–22-nt siRNAs (red) and 23–25-nt siRNAs (black) surrounding imprinted genes targeted by *DME* in WT (**b**) and *psd-13* (**c**) pollen. **d**–**g**, Images of representative seeds from crosses between WT Col-0 female and *osd1* diploid pollen (**d**), double-mutant *osd1*;*psd-13*^−/−^ diploid pollen (**e**), double-mutant *osd1*;*psd-13*^+/−^ diploid pollen (**f**) and double-mutant *osd1;nrpd1a-3* diploid pollen (**g**). *NRPD1*a encodes the large subunit of Pol IV. **h**, Frequency of aborted seeds from the same crosses. **i**, Model for transport of small RNA during pollen development. As the pollen matures, 21–22-nt easiRNAs and 24-nt siRNAs are produced by Pol IV in the VN and pseudouridylated along with some miRNAs. Pseudouridylated miRNAs and easiRNAs are exported by PSD (Exportin-t) into sperm cells, where they are loaded onto AGO1, AGO5 and AGO9, while unmodified miRNAs are transported by HST (Exportin-5) and loaded onto AGO5 and AGO1. Pseudouridylated sperm cell easiRNAs mediate dose-dependent lethality (the triploid block) by targeting MEGs in the seed. **j**, mCherry::AGO9 fusion protein expressed from the *AGO9* promoter (*pAGO9*) in developing pollen. DAPI was used to stain nucleic acids (blue). AGO9 is present in the VN at the bicellular stage but is enriched in the sperm cells in mature pollen. **k**, AGO9 IP from WT and *psd*-13 pollen shows increased binding of VN-derived 20–22-nt siRNAs and decreased binding of 23–25-nt sperm-cell-derived siRNAs to AGO9 in *psd*-13. Numbers on bars indicate the number of TEs analyzed; error bars indicate the s.e.m. *P* = <0.0001, 0.99, 0.99 and <0.0001, determined by one-way ANOVA with Bonferroni correction for multiple comparisons. In **a**,**k**, *P < 0.05, **P < 0.01 and ***P < 0.001. NS, not significant. Source data

easiRNAs from pollen target imprinted genes in *Arabidopsis*^9,11^. Imprinted genes in plants are expressed in the seed from either the maternal or the paternal genome and are known as maternally expressed and paternally expressed genes (MEGs and PEGs), respectively. *DEMETER* (*DME*) encodes a DNA glycosylase that is expressed in the endosperm and in the pollen VN, where it removes DNA methylation from TEs upstream and downstream of imprinted genes, resulting in parent-of-origin expression^47^. We, therefore, examined Ψ in siRNAs from DME targets and imprinted genes. We detected Ψ in 21–22-nt easiRNAs and 24-nt siRNAs from DME targets (Fig. 4a) and from TEs surrounding MEGs and PEGs (Extended Data Fig. 7m). Pseudouridylation of siRNAs from upstream and downstream of DME targets was lost in *psd-13* mutant pollen (Fig. 4a) and there was a striking depletion of easiRNAs from the promoters of DME target genes in *psd* sperm cells relative to WT (Fig. 4b,c). Ψ loss was more significant in MEGs than PEGs (*P* < 0.05; Extended Data Fig. 7m) and also affected sperm cell localization of siRNAs from both MEGs and PEGs, with 20–22-nt siRNAs depleted and 23–25-nt siRNAs enriched in *psd-13* sperm cells compared to WT (Extended Data Fig. 7n).

Pol IV-dependent 21–22-nt easiRNAs and miR845b mediate the triploid block, a dose-dependent epigenetic phenomenon whereby *Arabidopsis* seeds abort during development if they inherit an excess of paternal genomes from diploid instead of haploid pollen^9–11^. As 21–22-nt Pol IV-dependent easiRNAs are lost from sperm cells in *psd-13* pollen, this suggests that *psd-13* might rescue triploid seed abortion^9^. As expected, we observed ~90% seed abortion when WT Columbia plants were crossed with *omission of second division 1 (osd1)* mutants, which have diploid pollen (Fig. 4d). However, when *osd1*;*psd-13*^−/−^ diploid pollen was applied, this resulted in only ~10% seed abortion, similar to when *osd1*;*nrpd1a* diploid pollen was applied, which is deficient in the large subunit of Pol IV (NRPD1; Fig. 4e–h). Levels of miR845a and miR845b were not affected in *psd-13* mutant pollen and were not responsible for rescuing the triploid block (Extended Data Fig. 7o)^9^. Importantly, *osd1;psd-13*^+/−^ pollen parents gave rise to 50% aborted seeds (Fig. 4f,h). One possible explanation is that *psd* acts gametophytically within the pollen grain itself, supporting the observation that VN-derived 21–22-nt easiRNAs but not sperm-cell-derived small RNAs are required for the triploid block^44^.

Exportin-t is, therefore, essential for the triploid block and for pseudouridylation of easiRNAs. This implicates Ψ as a key modification in cell–cell transport of small RNAs in the germline by Exportin-t (Fig. 4i) and for epigenetic inheritance in the form of the triploid block. Cytoplasmic connections between the VN and sperm cells are thought to be involved^6,8^, consistent with a role for nucleocytoplasmic exportin. AGO5 is found in these ‘male germ units’ (ref. ^34^) and AGO5 is also required at least in part for the triploid block^35^. AGO9 has a similar localization (Fig. 2d,e) and has previously been associated with siRNA movement in pollen^48,49^. We, therefore, examined AGO9 localization using an mCherry N-terminal fusion^50^ and found that AGO9 was first localized in the VN at the bicellular stage but then moved to the sperm cells by the tricellular stage (Fig. 4j). This localization is consistent with a role for AGO9 before and after siRNA transport as previously proposed^48,51^. We, therefore, performed IP of small RNAs from WT and *psd* pollen using antibodies to AGO9. Remarkably, AGO9-associated 21–22-nt easiRNAs were substantially more enriched in *psd* mutants than in WT (Fig. 4k). One interpretation is that easiRNAs are trapped in the VN in *psd* mutants, where they are subsequently loaded onto AGO9.

### Ψ is found in mammalian germline piRNAs

**Extended Data Fig. 8:**
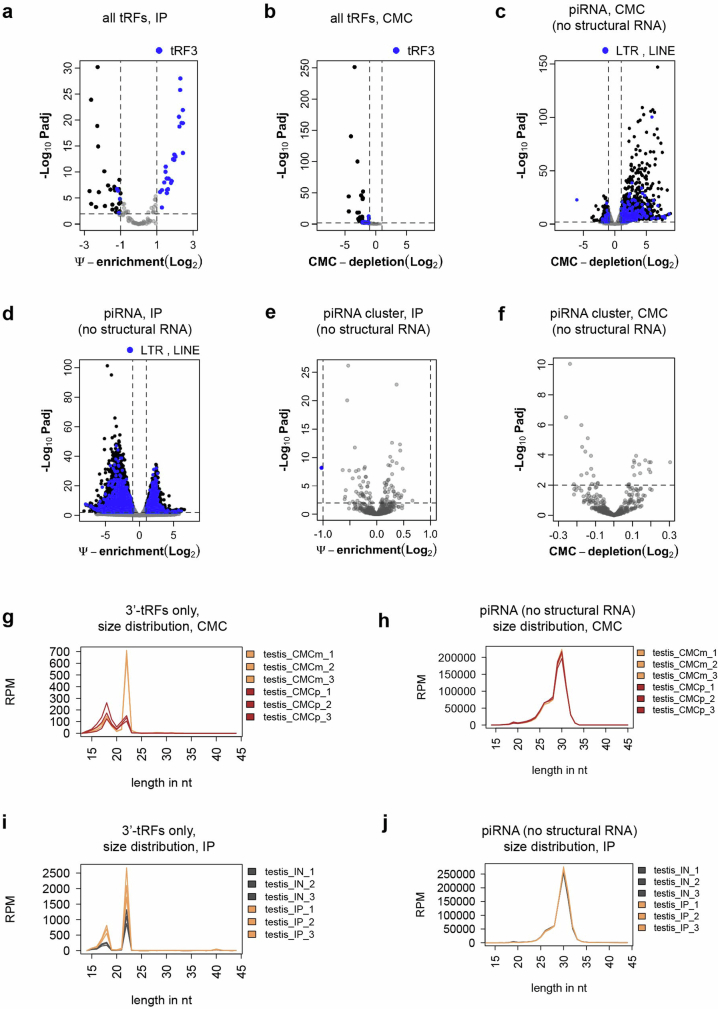
Pseudouridine in piRNAs and tRNA fragments (tRFs) from mouse testis. **(a, b)** Ψ detection by Ψ-IP (**a**) and CMC-depletion (**b**) of small RNA from testes of 8 weeks-old male mice. Dots correspond to tRF counts per tRNA isoacceptor. Differential expression (fold change) and statistical significance were calculated using the DESeq2 package^61^. Statistically significant (p-adj. <0.01, log2 fold change > |1|) enriched or depleted tRFs are in black, 3’-tRFs are colored blue; grey dots mark tRFs without significant enrichment/depletion. **(c)** Volcano plot of read counts per piRNA sequence in testes (8 weeks old males) by CMC-depletion and sequencing. Black: significant fold changes (p-adj. <0.01, log2 fold change > |1|) as per DESeq2 analysis; grey: not significant; blue: significantly enriched or depleted piRNAs overlapping LTR and LINE transposon sequences. (**d**) Volcano plot of read counts per piRNA sequence by Ψ-IP. Black: significant fold changes (p-adj. <0.01, log2 fold change > |1|) as per DESeq2 analysis; grey: not significant; blue: significantly enriched or depleted piRNAs overlapping LTR and LINE transposon sequences. (**e,f**) Volcano plot of read counts per piRNA cluster using Ψ-IP (**e**) or CMC-depletion (**f**) and DESeq2 for statistical analysis. **(g,h)** Size distribution of 3’-tRFs (**g**) and piRNA (**h**) sequences (without structural RNAs) using CMC-depletion and sequencing. **(i, j)** Size distribution of 3’-tRFs (**i**) and piRNA (**j**) sequences (without structural RNAs) using Ψ-IP and sequencing. RPM: reads per million mapped reads. Source data

easiRNAs and phasiRNAs in the plant germline have similar functions to piRNAs in the mammalian germline; thus, we asked whether high levels of Ψ were conserved in piRNAs. We used Ψ-IP and CMC depletion to detect Ψ in small RNAs from 8-week-postpartum mouse testes. We found that 3′-tRNA fragments (tRFs) were enriched by Ψ-IP (Extended Data Fig. 8a) and depleted by CMC treatment (Extended Data Fig. 8b). Importantly, many piRNAs from TEs, including from long interspersed nuclear elements (LINEs) and LTR retroelements, were strongly enriched for Ψ (Extended Data Fig. 8c,d). Up to 3% of piRNAs were differentially enriched for Ψ, although many more were immunoprecipitated and, hence, potentially pseudouridylated (Extended Data Fig. 8d). No individual piRNA cluster was significantly enriched or depleted, suggesting sequence rather than locus specificity (Extended Data Fig. 8e,f). The 3′-tRFs that were depleted by CMC treatment were 22 nt in size as expected for 3′-tRFs containing Ψ-55 from mature tRNAs (Extended Data Fig. 8g), while piRNAs were mostly 30 nt in size as expected (Extended Data Fig. 8h). The same small RNA sizes and classes were detected by Ψ-IP (Extended Data Fig. 8i,j). Thus, not only 3′-tRFs but also piRNAs and miRNAs in mammals are pseudouridylated. Intriguingly, 5′-tRFs are also pseudouridylated and have been reported to be inherited^52,53^.

## Discussion

Thus, germline small RNAs in plants and mammals are heavily pseudouridylated, which, at least in plants, depends on Exportin-t. This is true of both VN-derived and sperm-cell-derived small RNAs in pollen, implicating nucleocytoplasmic shuttling in pseudouridylation, as previously proposed^54^. Intriguingly, *PSD* is also required for the transport of VN-derived easiRNAs to sperm cells and for the triploid block. Therefore, Ψ and/or intercellular transport is required for epigenetic inheritance in the form of the triploid block. Why are germline small RNAs so heavily modified in both plants and mammals? An intriguing possibility is that modifications of RNA in the germline may avoid viral surveillance systems after fertilization, which could otherwise recognize inherited small RNAs as ‘nonself’ (ref. ^55^). RTL1 is such a viral surveillance mechanism and is induced strongly on viral infection^56^. While VN-derived easiRNAs are suppressed when RTL1 is expressed in the VN, they are not suppressed when RTL1 is expressed in sperm cells even though VN-derived easiRNAs accumulate in sperm cells^44^. An intriguing explanation might be that easiRNA pseudouridylation requires export from the VN and RTL1 cannot recognize these easiRNAs once they are pseudouridylated.

## Methods

### Cell lines and tissues

NIH/3T3 (American Type Culture Collection (ATCC), CRL-1658) cells were cultured in DMEM (Invitrogen) supplemented with 10% FBS and 1% penicillin, streptomycin and glutamine. Cells tested negative for *Mycoplasma* contamination. Testes were harvested from C57Bl/6 mice at 8 weeks postpartum and flash-frozen in liquid nitrogen. All animal procedures and studies were conducted in accordance with the Institutional Animal Care and Use Committee at Cold Spring Harbor Laboratory.

### Plant growth conditions

*Arabidopsis* plants were grown under long-day conditions at 22 °C. Seeds were always surface-sterilized with sodium hypochlorite, sown on Murashige and Skoog medium and stratified for 3 days at 4 °C. Seedlings were transplanted to soil 2 weeks after germination and grown under long-day conditions at 22 °C. The following mutants were used: *pus7*-1 (SALK_098773), *pus10*-1 (SALK_091360) and *nrpd1a*-3 (SALK_128428). Homozygous mutants were identified using PCR with primers specific to the predicted site of T-DNA integration (Supplementary Table 11). The *psd*-13 and *hst*-6 alleles were previously identified^39,57^. The *osd1*-3 mutant was kindly provided by R. Mercier (Max Planck Institute for Plant Breeding Research).

### Yeast two-hybrid screening

The two-hybrid screen was performed by Hybrigenics using an *Arabidopsis thaliana* Col-0 library (ATFO) prepared from young flowers using an in-house cDNA clone of AGO3 as bait. Seven candidate proteins were tested by BiFC, leading to the identification of AT5G08180, which encodes NHP2, as a high-confidence interactor. This was subsequently confirmed by MS.

### BiFC

BiFC was performed using pBiFC-2in1 vectors^58^. AGO3 and NHP2 were fused in all orientations (NN, CN, NC, CC or only NN and CC complemented), while other AGO proteins were fused only in NN configuration. Tobacco leaves were infiltrated essentially as previously described^59^. Briefly, *Agrobacterium* cultures harboring pBiFC vectors were grown overnight before subculturing in 50 ml of Luria–Bertani broth with antibiotics and 20 µM acetosyringone. Cultures were pelleted by centrifugation and resuspended in infiltration buffer (10 mM MES, 10 mM MgCl_2_ and 100 µM acetosyringone). Leaves of ~3-week-old *Nicotiana benthamiana* plants were infiltrated on the abaxial surface using a syringe without a needle. Then, 3 days after infiltration, leaf disks were removed and imaged using a Zeiss LSM780 confocal microscope. Primers for cloning AGO proteins and NHP2 are detailed in Supplementary Table 11.

### Construction of viable *dkc1* mutants in *Arabidopsis*

First, the *AtNAP57 (DKC1)* genomic sequence was amplified and cloned into the pENTR vector. The *ABI3* promoter sequence was amplified and inserted using the NotI restriction site. The *pABI3:AtNAP57* cassette was transferred to the pGWB501 vector using the Gateway cloning system and used for *Agrobacterium*-mediated, floral dip *Arabidopsis* transformation of *nap57*^−/+^ (SALK_031065) heterozygous plants. Homozygous transformant lines were obtained by three rounds of hygromycin resistance selection followed by verification of *nap57/nap57* genetic background by PCR.

### Co-IP and MS analyses

For each co-IP, 0.6 g of tissue from GFP-tagged NHP2 plants was used. The powder was extracted for 30 min in 1.5 ml of lysis buffer (50 mM Tris-HCl pH 8.0, 50 mM NaCl, 1% Triton X-100 and 1× cOmplete EDTA-free protease inhibitor (Roche)). After removal of cell debris by centrifugation (twice for 10 min at 16,000*g*, 4 °C), the cleared supernatants were incubated for 30 min with anti-GFP antibodies coupled to magnetic microbeads (µMACS GFP isolation kits, Miltenyi). Beads were loaded on magnetized MACS separation columns equilibrated with lysis buffer and washed four times with 300 µl of washing buffer (50 mM Tris-HCl pH 7.5 and 0.1% Triton X-100). Samples were eluted in 100 µl of prewarmed elution buffer (Miltenyi). Control IPs were performed using Col-0 plants and GFP antibodies. Eluted proteins were digested with sequencing-grade trypsin (Promega). Peptide mixtures were separated using Evosep One (Evosep Biosystems) chromatography coupled to Orbitrap Exploris 480 (Thermo Fisher Scientific) at the Institute of Biochemistry and Biophysics, Polish Academy of Sciences. Data were searched against the TAIR10 database. Peptides were identified with the Mascot algorithm (Matrix Science). The total number of MS/MS fragmentation spectra was used to quantify each protein from three replicates. The statistical analysis based on spectral counts was performed using a homemade R package that calculates fold change and *P* values using the quasi-likelihood negative binomial generalized log-linear model implemented in the edgeR package^60^. *P* values were adjusted using the Benjamini–Hochberg method from the stats R package. The size factor used to scale samples was calculated according to the DESeq2 normalization method^61^.

### miRNA precursor pulldown with GFP-tagged NHP2

Anti-GFP antibodies were used to immunoprecipitate GFP-tagged NHP2 from three independent transgenic lines expressing GFP-tagged NHP2 under a native promoter, along with WT plants as a negative control. RNA was converted into cDNA libraries using the NEBNext Ultra II directional RNA library prep kit for Illumina. Libraries were sequenced with Nextseq HO 100-bp paired-end reads. Adaptors and unpaired reads were removed using Trimmomatic and files were analyzed using Salmon software. Obtained transcripts per million values for each transcript were adjusted with 0.1 and 0.01 for snRNA or snoRNA and pri-miRNA or pre-miRNA, respectively, to avoid dividing by zero in the case of nondetected transcripts. IP values were normalized against input and the fold change was calculated for GFP-tagged NHP2 versus the negative control (GFP IP in WT plants). Reproducibly enriched RNAs in NHP2 IP had a fold change ≥ 1.2 in at least two lines.

### RNA extraction of mammalian cells

Cells were lysed in Qiazol (Qiagen) and total RNA was purified using the miRNEasy mini kit (Qiagen) according to the manufacturer’s instructions. Tissues were lysed in Qiazol (Qiagen) and total RNA was purified using Tissuelyser II (Qiagen) as per the manufacturer’s instructions. Small RNAs (<200 nt) were size-fractionated using the RNA Clean and Concentrator 5 column kits (Zymo) as per the manufacturer’s instructions. Testes of 8-week-old mice were ground under liquid nitrogen and RNA was extracted using Trizol (Thermo Fisher Scientific) according to the manufacturer’s instructions but using 80% ethanol during the wash step.

### Dot blot

For dot-blot analysis, input RNA or RNA immunoprecipitated with either anti-Ψ or isotypic nonspecific antibodies was spotted onto a nitrocellulose membrane and crosslinked under ultraviolet light at 254 nm (120 mJ cm^−2^). The membranes were blocked in Denhart’s solution (1% Ficoll, 1% polyvinylpyrrolidone and 1% BSA; Thermo Fisher Scientific) for 1 h at room temperature and incubated with Ψ antibody for 1 h at room temperature. The signal was detected using horseradish peroxidase-conjugated secondary antibodies and enhanced chemiluminescence (GE Healthcare) and developed on a Chemidoc MP machine (BioRad).

### Ψ-IP

Small RNAs were isolated from 0.01–0.1 mg of total RNA, first by separating <200-nt RNAs with the Zymo Clean and Concentrator kit and subsequently by gel-extracting 20–30-nt RNAs on a 15% polyacrylamide gel. Small RNAs were eluted from the crushed gel slice overnight in 500 µl of RNA elution buffer (0.3 M NaCl and 0.5 mM EDTA). RNA was resuspended in 50 µl of water, typically containing ~4–40 ng µl^−1^ RNA. Resuspended RNA was mixed with 200 µl of IPP buffer (150 mM NaCl, 0.1% NP-40 and 10 mM Tris-HCl pH 7.4 + 10 U per ml of RNAse inhibitor) and 5 µg of mouse monoclonal Ψ antibody (D347-3, MBL Intl.). Samples were incubated for 2 h at 4 °C with rotation before adding 50 µl of sheep antimouse IgG dynabeads washed in IPP buffer (Invitrogen). The unbound fraction was retained and the beads were washed four times with IPP buffer. To prepare RNA from the unbound fraction, 400 µl of binding buffer from the Zymo Clean and Concentrator kit was added and RNA was run through the column according to manufacturer’s instructions. To elute bound plant siRNA from the beads, 150 µl of Trizol was added and incubated for 2 min before placing tubes on a magnetic rack. Trizol was transferred to a new tube and 30 µl of chloroform was added and mixed thoroughly. Samples were centrifuged for 10 min at maximum speed and the aqueous phase was pipetted into a new tube with an equal volume of 100% ethanol. Mouse small RNAs were eluted using 6.7 mM Ψ triphosphate in IPP buffer with RNase-OUT (Thermo Fisher Scientific) for 30 min at 37 °C. Samples were then run through a Zymo Clean and Concentrator kit according to the manufacturer’s instructions and eluted in a final volume of 8 µl. Libraries were prepared using a NEBNext small RNA-seq kit (New England Biolabs) or NextFlex small RNA-seq kit (Perkin Elmer) according to the manufacturer’s instructions, with the same temperature modifications as the CMC-treated samples. Libraries were pooled and run on NextSeq 500, HiSeq 4000 or MiSeq high-throughput sequencing systems (Illumina).

### Nucleoside analysis of RNA

RNA samples were incubated overnight with a 2× nuclease mix containing 62.5 U of Benzonase (Sigma-Aldrich), 5 U of Antarctic phosphatase (NEB) and 10 mU per µl of phosphodiesterase I from *Crotalus adamanteus* venom (Sigma-Aldrich) made up in a 5× digest buffer composed of 20 mM Tris-HCl pH 8, 20 mM MgCl_2_ and 100 mM NaCl. Samples were then further purified to remove protein by filtration at 17,000*g* for 30 min through Amicon Ultra-0.5 spin columns (Sigma-Aldrich) with a 30-kDa molecular weight cutoff^62^. Samples were then analyzed using a Q Exactive HF Orbitrap MS instrument (Thermo Fisher Scientific). Briefly, 2 µl of digested sample was loaded onto a 100-Å, 1.8-µm, 2.1 × 100-mm ACQUITY UPLC HSS T3 column (Waters) in an aqueous buffer consisting of 0.1% MS-grade formic acid in water and acetonitrile (98:2) at a constant flow of 0.3 ml min^−1^. A gradient of organic solvent was used to facilitate separation and elution as follows: from 2 to 8 min, the percentage of organic solvent (0.1% formic acid in acetonitrile) increased from 2% to 10%; from 8 to 13 min, the gradient increased from 10% to 98% organic solvent, where it was held for 1 min before returning to 2% organic to re-equilibrate the column for the next sample. Samples were read in full scan positive mode from 100 to 600 *m*/*z* and data were analyzed using Xcalibur software (Thermo Fisher Scientific). All nucleosides were compared to exogenous standards, which allowed the interpolation of concentrations of sample nucleosides. Isobaric nucleosides were differentiated on the basis of their retention times as compared to pure commercial standards. As an example, Ψ had a retention time of 1.55 min in this protocol, whereas uridine eluted at 3.05 min.

### RT–qPCR

Cells were lysed in Qiazol (Qiagen) and total RNA was purified using the miRNEasy mini kit (Qiagen) according to the manufacturer’s instructions. For mRNA detection, 1 μg of purified total RNA was reverse-transcribed using the high-capacity cDNA RT kit (Applied Biosystems). For specific miRNA quantification, we size-fractionated small RNAs (<200 nt, containing pre-miRNAs) using RNA Clean & Concentrator 5 column kits (Zymo), as per the manufacturer’s instructions. miRNAs were reverse-transcribed with the miScript II RT kit (Qiagen). Primers were designed to anneal on the 5′ or 3′ arm of the miRNA of interest (Supplementary Table 11). miRNAs were quantified using Fast SybrGreen PCR mastermix (Applied Biosystems) according to the manufacturer’s instructions.

### CMC treatment and small RNA-seq

For each pair of libraries, 4 µg of *Arabidopsis* inflorescence RNA, prepared using the Zymo Quickzol RNA kit, was ligated to the 3′ adapter supplied in the NEB NextSeq kit in a 40-µl reaction. After ligation, samples were divided in two and subjected to CMC or mock treatment. Then, 80 µl of 0.5 M CMC in BEU buffer (7 M urea, 4 mM EDTA and 50 mM bicine pH 8) was added to 20 µl of sample and incubated at 37 °C for 30 min. Mock-treated samples were incubated with 80 µl of BEU buffer. After CMC or mock treatment, 25 µl of 3 M sodium acetate, 3 µl of glycogen and 1 ml of 100% ethanol were added and samples were incubated for 1 h at −80 °C. Samples were centrifuged at 4 °C for 30 min and the resulting pellets were washed with 80% ethanol, followed by another 1 h at −80 °C. Samples were centrifuged at 4 °C for 30 min and the ethanol was removed. Pellets were allowed to dry for 5–10 min before the addition of 50 µl of freshly prepared 0.05 M carbonate buffer (pH 10.4) containing 4 mM EDTA. Samples were incubated at 37 °C for 3 h and subsequently cleaned using the Zymo RNA Clean and Concentrator kit, eluting in a final volume of 25.5 µl. After cleaning, libraries were prepared according to instructions in the NEBNext small RNA-seq kit (New England Biolabs) or Nextflex Small RNA-Seq Kit v3 (Perkin Elmer), beginning with annealing of the RT primer, with some modifications. Primer annealing was performed at 65 °C for 5 min, followed by 37 °C for 15 min and 25 °C for 15 min. cDNA synthesis was carried out at 42 °C. After PCR amplification, libraries were cleaned using the Qiagen PCR cleanup kit and run on a 6% acrylamide gel. Bands of ~140 nt were cut from the gel, crushed and eluted for 2 h in DNA elution buffer followed by ethanol precipitation as outlined in the manufacturer’s instructions. Libraries were pooled and run on NextSeq or MiSeq high-throughput sequencing systems (Illumina) at the Cold Spring Harbor Laboratory sequencing facility.

### CMC/Mn^2+^ treatment and sequencing

For high-throughput siRNA mismatch analysis, 2 µg of total *Arabidopsis* RNA was treated as described above for CMC siRNA-seq libraries with some modifications. The Nextflex Small RNA-Seq Kit v3 (Perkin Elmer) was used for all samples. The RT step was modified to use SuperScript II (Thermo Fisher Scientific) and a custom 5× first-strand buffer containing MnCl_2_ (250 mM Tris-HCl pH 8.3, 375 mM KCl and 30 mM MnCl_2_). For pri-miRNA mismatch analysis, 10 µg of total RNA was treated in the same way as for siRNA mismatch analysis. The obtained cDNA was used for amplification with pri-miRNA-specific primers. PCR products were gel-purified and cloned into pGEM-T Easy (Promega) for Sanger sequencing. The same number of clones was sequenced for corresponding CMC^+^ and CMC^−^ samples.

### Northern blotting

First, 50 µg of total RNA was treated with 0.5 M CMC in BEU buffer (typically 25 µl of RNA in 200 µl of buffer). Samples were cleaned and prepared in the same way as for small RNA-seq, except that RNA < 200 nt was prepared using the Zymo RNA Clean and Concentrator kit to enrich small RNA in a very low volume. Samples were briefly denatured at 65 °C for 2 min (higher temperatures were avoided as these can remove the CMC adducts) before loading onto a 15% denaturing polyacrylamide TBE gel. CMC^+^ and CMC^−^ samples were treated identically except for the addition of CMC. Untreated RNA was loaded directly onto the gel after denaturation. Gels were run until the bromophenol blue band reached the bottom. RNA was transferred to a Hybond NX membrane using a semidry electroblotter for 30 min. Crosslinking was performed with EDC^63^. Membranes were blocked using digoxigenin (DIG) Easy Hyb buffer (Roche) followed by incubation with 10 pmol ml^−1^ 5′/3′ DIG-labeled LNA probe (Exiqon) in Easy Hyb buffer at 65 °C overnight. Probes were detected using Roche DIG wash and block buffer set, anti-DIG antibody and CDP star reagent according to the manufacturer’s instructions.

### IP of AGO9

IP of AGO9 from *Arabidopsis* pollen was performed as previously described^35^. Pollen was collected from open *Arabidopsis* flowers as described above before grinding in liquid nitrogen and homogenizing in 5 ml g^−1^ extraction buffer for 1 h at 4 °C (50 mM Tris-HCl pH 7.5, 150 mM NaCl, 10% glycerol, 0.2% NP-40, 5 mM MgCl_2_ and 5 mM DTT, containing one tablet per 10 ml of protease inhibitor cocktail (Roche)). Cell debris was removed by centrifugation at 14,000*g* at 4 °C for 20 min and the supernatant was recovered. Protein extract was precleared by incubation with 10 μl of protein A Dynabeads (Invitrogen) at 4 °C for 30 min. Supernatant was recovered by centrifugation at 14,000*g* at 4 °C for 1 min. Precleared extracts were incubated with anti-AGO9 antibody (1:100 dilution) and 30 μl of protein A Dynabeads at 4 °C overnight. The immunoprecipitate was pelleted and washed three times (15 min each in rotator shaker) in extraction buffer. Small RNAs from the purified AGO9 complex were isolated using Ambion columns. Small RNA was resolved on a 12.5% denaturing PAGE 7M Urea gel and stained with SYBR gold (Invitrogen). For cloning, gel slices within the range of 18–30 nt were excised and the RNAs were eluted and purified as described above. Small RNAs were sequenced by Fasteris (Switzerland).

### Flow cytometry and staining of *Arabidopsis* pollen

Flow cytometry and Alexander staining was performed as previously reported^64^. Pollen was collected in 1.5-ml Eppendorf tubes by vortexing open flowers in pollen extraction buffer (PEB; 10 mM CaCl_2_, 2 mM MES, 1 mM KCl and 1% H_3_BO_3_, 10%) for 3 min 47 s, followed by filtration through a 30-µm mesh (Partec/Sysmex) and centrifugation at 5,000*g* for 1 min. Pollen was suspended in 50 µl of PEB, immediately frozen in liquid nitrogen and stored at −80 °C until RNA extraction. Open flowers were collected into a 50-ml falcon tube, frozen in liquid nitrogen and stored at −80 °C for storage. The pollen enriched fraction was isolated with the procedure described by Borges et al.^64^ using sperm extraction buffer (SEB). Intact pollen grains were sorted with FACS using a S3e Cell Sorter (BioRad). To the obtained pollen sample in a 1.5-ml tube, 100 μl of acid-washed glass beads (425–600 μm; Sigma-Aldrich) were added and vortexed continuously at medium speed for 1 min to break mature pollen grains. The sample was transferred to a new tube and the volume of the sample was made up to 1 ml with SEB. Then, 1 µl of SYBR green was added for staining. The sample was readied for FACS of sperm cells according to Borges et al.^64^. RNA from the sperm cells fraction was isolated with Trizol, treated with DNase I and used for small RNA library preparation (NextFlex Small RNA Kit, Perkin Elmer). Pollen staining was performed following Alexander’s staining protocol^65^. Only viable pollen grains took up the stain.

### Bioinformatic analysis of rRNA coverage

Adaptors were trimmed from reads, converted to FASTA files and collapsed using the FASTX toolkit. Trimmed reads between 15 and 30 nt in length were mapped to the *Arabidopsis* genome (TAIR10) using Bowtie^66^ allowing mismatches and multimapping. Coverage across the rRNA locus on chromosome 2 (position 3706–9521) was calculated using BEDTools^67^. Coverage within a sample was normalized to the total amount of coverage across the locus and the log_2_(fold change) between paired samples (IP/unbound and CMC/mock) was calculated and averaged between technical replicates. Sites of predicted pseudouridylation were taken from published datasets and the average log_2_(fold change) surrounding these sites was calculated^68,69^.

### Bioinformatic analysis of CMC/Mn^2+^-treated *Arabidopsis* samples

For adaptor trimming and size selection of reads (18–42 nt), Cutadapt was used^70^. Terminal deletions in CMC^+^ samples were detected using DeAnnIso^71^ or miRMOD^72^. For internal deletion detection, reads were aligned to the *Arabidopsis* genome (TAIR10) using BWA^73^ and further processed using SAMtools^74^ bam-readcount and AWK homemade scripts. For the identification of Ψ-modified RNA bases in the CMC/Mn^2+^ protocol, the following criteria were used: at least five reads for a particular miRNA and at least 1.5 CMC^+^/CMC^−^ fold change for the ratio between reads with mismatch and all reads for a particular miRNA.

### Bioinformatic analysis of *Arabidopsis* siRNA and miRNA

Adaptors were trimmed from reads, converted to FASTA files and collapsed using the FASTX toolkit. Trimmed reads between 20 and 25 nt in length were mapped to the *Arabidopsis* genome (TAIR10) using Bowtie, allowing mismatches and discarding unmapped reads. Individual sequences with an average of >10 reads per sample were selected and analyzed using DESeq2 (ref. ^61^), treating CMC^+^/mock and IP/unbound fractions as pairs. Mature miRNA sequences from miRBase (version 21) were extracted from these datasets. Sequences mapping to tRNA, snoRNA, snRNA or rRNA were removed for analysis of 5′ and 3′ bias. UNUAR-containing and 3ʹCCA tRFs were identified by extracting reads mapping to tRNAs and selecting those containing the UNUAR motif or 3ʹCCA; these sequences were extracted from the DESeq2 output for plotting.

### Bioinformatic analysis of mouse miRNA

The mouse small RNA-seq data from NIH/3T3 cells were analyzed with the SeqImp pipeline configured with the mm10 assembly of the *Mus musculus* genome, the transcriptome annotation from Ensembl (version 89) and the miRbase annotation of microRNAs (version 21). The pipeline was executed with the default mouse miRNA configuration and default parameters, specifying the sequence of the 3′ adapter used for library preparation (AGATCGGAAGAGCACACGTC). The miRNA quantifications obtained with SeqImp were further analyzed in R/Bioconductor with the DESeq2 package^61^. Briefly, counts were normalized to the library size using the median ratio method as implemented in the ‘estimateSizeFactors’ function. Variance was estimated with the ‘estimateDispersions’ function and statistical testing for enrichment was performed with the Wald test as implemented in the nbinomWaldTest function. *P* values were corrected for multiple hypothesis testing with the Benjamini–Hochberg procedure.

### Analysis of TE-derived siRNA

Collapsed reads 18–30 nt long were mapped to the TAIR10 genome, allowing multimapping but no mismatches, using Bowtie. Quantification of siRNA mapping to each TE was performed as described previously^9^. Reads mapping to TE families were aggregated and the average log_2_(fold change) between paired samples was calculated. The mean of all technical replicates (generated by separate CMC/mock or IP/unbound library preparations on the same RNA) for any given biological replicate was taken before calculating the mean of all biological replicates for each tissue (inflorescence or pollen) and treatment (CMC or IP) combination.

### Metaplots

Uncollapsed reads 20–25 nt in length were aligned to the *Arabidopsis* (TAIR10) genome using Bowtie, allowing mismatches but only a single mapping event per read. Mapping files from the same treatment (that is, CMC^+^, CMC^−^, IP and unbound) were merged after normalization (as above) to enable the production of a single representative plot. Coverage was calculated using BEDTools^67^. Metaplots were produced using the Versatile Aggregate Profiler^75^ with a window size of 25 and a smoothing window of 6 for TE analysis and a window size of 50 and smoothing window of 12 for DME target analysis. For comparison between *psd-13* mutants and WT, metaplots of three biological replicates were created and the mean enrichment was plotted.

### Bioinformatic analysis of mouse testis small RNA

Reads were clipped for Illumina Truseq (AGATCGGAAGAGCACACGTCTGAAC) or Perkin Elmer Nextflex Small RNA adapter sequences (N(4)TGGAATTCTCGGGTGCCAAGG) using Cutadapt. Reads were required to be 14–44 nt and have a quality score of at least Q20 over 90% of their length. tRFs were analyzed as described previously^76^ using all tRNA sequences included in the current University of California, Santa Cruz (UCSC) repeat annotation (ftp://hgdownload.soe.ucsc.edu/goldenPath/mm10/database/rmskOutCurrent.txt.gz). Reads that were not tRFs were mapped to the UCSC mm10 genome sequence using Bowtie 2, filtered for 0–3 mismatches (to accommodate mismatches because of RNA modifications) using SAMtools and BAMtools and intersected with the UCSC repeat annotation using BEDTools. Repeat and nonrepeat reads were further intersected with mm10 mouse piRNA clusters (https://www.smallrnagroup.uni-mainz.de/piCdb/) to result in piRNA reads that overlapped transposon sequences, structural RNAs or neither. Raw counts of individual piRNA sequences were analyzed for Ψ enrichment (enriched in IP, depleted in CMC-treated libraries) using DESeq2 (ref. ^61^). Similarly, raw counts per piRNA cluster were analyzed using DESeq2 but each cluster was required to have at least an average of 100 raw reads (per IP or per CMC-untreated sample). Read counts that were used as input for DESeq2 analysis were from three replicates of each condition (input, IP, CMC-untreated and CMC-treated). The log_2_(fold change) was plotted against the −log10(adjusted *P* value) using R.

### Reporting summary

Further information on research design is available in the Nature Portfolio Reporting Summary linked to this article.

## Online content

Any methods, additional references, Nature Portfolio reporting summaries, source data, extended data, supplementary information, acknowledgements, peer review information; details of author contributions and competing interests; and statements of data and code availability are available at 10.1038/s41594-024-01392-6.

## Supplementary information

Reporting Summary

Supplementary Table 1 Supplementary Tables 1–11.

## Source data

Source Data Fig. 1 Unprocessed northern blots.

Source Data Fig. 1 Statistical source data.

Source Data Fig. 2 Statistical source data.

Source Data Fig. 3 Statistical source data.

Source Data Fig. 4 Statistical source data.

Source Data Extended Data Fig. 1 Statistical source data.

Source Data Extended Data Fig. 2 Statistical source data.

Source Data Extended Data Fig. 3 Statistical source data.

Source Data Extended Data Fig. 4 Statistical source data.

Source Data Extended Data Fig. 5 Statistical source data.

Source Data Extended Data Fig. 6 Statistical source data.

Source Data Extended Data Fig. 7 Statistical source data.

Source Data Extended Data Fig. 8 Statistical source data.

## Data Availability

Data were deposited to the Gene Expression Omnibus under accession numbers GSE229986, GSE230359, GSE230228 and GSE222751. Proteomic data were deposited to the PRIDE repository under accession number PXD042662. Source Data are provided with the paper.
