# Supplementary material for: Pseudouridine guides germline small RNA transport and epigenetic inheritance
Source: Nat Struct Mol Biol. 2024 Sep 6;32(2):277–86. doi: 10.1038/s41594-024-01392-6 (PMC11832342; doi:10.1038/s41594-024-01392-6)
Supplement: Supplementary file 1 — Reporting Summary [file 41594_2024_1392_MOESM1_ESM.pdf]

nature portfolio

Corresponding author(s): Robert A Martienssen (martiens@cshl.edu)

Last updated by author(s): Apr 8, 2024

Reporting Summary

Nature Portfolio wishes to improve the reproducibility of the work that we publish. This form provides structure for consistency and transparency in reporting. For further information on Nature Portfolio policies, see our Editorial Policies and the Editorial Policy Checklist.

Statistics

For all statistical analyses, confirm that the following items are present in the figure legend, table legend, main text, or Methods section.

n/a	Confirmed	
☐	☒	The exact sample size (*n*) for each experimental group/condition, given as a discrete number and unit of measurement
☐	☒	A statement on whether measurements were taken from distinct samples or whether the same sample was measured repeatedly
☐	☒	The statistical test(s) used AND whether they are one- or two-sided<br>*Only common tests should be described solely by name; describe more complex techniques in the Methods section.*
☐	☒	A description of all covariates tested
☐	☒	A description of any assumptions or corrections, such as tests of normality and adjustment for multiple comparisons
☐	☒	A full description of the statistical parameters including central tendency (e.g. means) or other basic estimates (e.g. regression coefficient) AND variation (e.g. standard deviation) or associated estimates of uncertainty (e.g. confidence intervals)
☐	☒	For null hypothesis testing, the test statistic (e.g. *F*, *t*, *r*) with confidence intervals, effect sizes, degrees of freedom and *P* value noted<br>*Give P values as exact values whenever suitable.*
☐	☒	For Bayesian analysis, information on the choice of priors and Markov chain Monte Carlo settings
☐	☒	For hierarchical and complex designs, identification of the appropriate level for tests and full reporting of outcomes
☐	☒	Estimates of effect sizes (e.g. Cohen's *d*, Pearson's *r*), indicating how they were calculated

Our web collection on statistics for biologists contains articles on many of the points above.

Software and code

Policy information about availability of computer code

Data collection	All protocols are described in the Methods section of the manuscript.
Data analysis	All analysis are described in the Methods section of the manuscript. The following softwares were used: Bismark v0.22.3; Sickle v1.33; bowtie2 v2.4.1; Samtools v1.10; BEDtools v2.31.0; MACS2 v2.2.7.1; bonito v0.6.2; modkit v0.2.3; dorado v0.5.0; minimap2 v2.26-r1175; deeptools v3.5.0

For manuscripts utilizing custom algorithms or software that are central to the research but not yet described in published literature, software must be made available to editors and reviewers. We strongly encourage code deposition in a community repository (e.g. GitHub). See the Nature Portfolio guidelines for submitting code & software for further information.

Data

Policy information about availability of data

All manuscripts must include a data availability statement. This statement should provide the following information, where applicable:

- Accession codes, unique identifiers, or web links for publicly available datasets
- A description of any restrictions on data availability
- For clinical datasets or third party data, please ensure that the statement adheres to our policy

Sequence data generated for this study have been deposited in Gene Expression Omnibus with the accession codes GSE132005

Research involving human participants, their data, or biological material

Policy information about studies with human participants or human data. See also policy information about sex, gender (identity/presentation), and sexual orientation and race, ethnicity and racism.

Reporting on sex and gender	NA
Reporting on race, ethnicity, or other socially relevant groupings	NA
Population characteristics	NA
Recruitment	NA
Ethics oversight	NA

Note that full information on the approval of the study protocol must also be provided in the manuscript.

Field-specific reporting

Please select the one below that is the best fit for your research. If you are not sure, read the appropriate sections before making your selection.

☒ Life sciences ☐ Behavioural & social sciences ☐ Ecological, evolutionary & environmental sciences

For a reference copy of the document with all sections, see nature.com/documents/nr-reporting-summary-flat.pdf

Life sciences study design

All studies must disclose on these points even when the disclosure is negative.

Sample size	No sample-size calculations were performed for this study. Sample sizes were chosen to match or exceed standards in the field.
Data exclusions	No data was excluded from the analysis.
Replication	At least 2 biological replicates were performed for all experiments. The number of replicates is indicated for each experiment.
Randomization	Plants were grown in random order in the greenhouse, and were phenotyped in the same random order.
Blinding	Phenotyping was performed without prior knowledge of the genotype

Reporting for specific materials, systems and methods

We require information from authors about some types of materials, experimental systems and methods used in many studies. Here, indicate whether each material, system or method listed is relevant to your study. If you are not sure if a list item applies to your research, read the appropriate section before selecting a response.

Materials & experimental systems

n/a	Involved in the study	
☐	☒	Antibodies
☒	☐	Eukaryotic cell lines
☒	☐	Palaeontology and archaeology
☒	☐	Animals and other organisms
☒	☐	Clinical data
☒	☐	Dual use research of concern
☐	☒	Plants

Methods

n/a	Involved in the study	
☐	☒	ChIP-seq
☒	☐	Flow cytometry
☒	☐	MRI-based neuroimaging

Antibodies

Antibodies used	anti-H3K9me2 (ab1220, Abcam); anti-CENH3 (gift from Prof. S. Henikoff)
Validation	manufacturer tested antibody and/or validated in previous studies

Dual use research of concern

Policy information about dual use research of concern

Hazards

Could the accidental, deliberate or reckless misuse of agents or technologies generated in the work, or the application of information presented in the manuscript, pose a threat to:

No	Yes	
☒	☐	Public health
☒	☐	National security
☒	☐	Crops and/or livestock
☒	☐	Ecosystems
☒	☐	Any other significant area

Experiments of concern

Does the work involve any of these experiments of concern:

No	Yes	
☒	☐	Demonstrate how to render a vaccine ineffective
☒	☐	Confer resistance to therapeutically useful antibiotics or antiviral agents
☒	☐	Enhance the virulence of a pathogen or render a nonpathogen virulent
☒	☐	Increase transmissibility of a pathogen
☒	☐	Alter the host range of a pathogen
☒	☐	Enable evasion of diagnostic/detection modalities
☒	☐	Enable the weaponization of a biological agent or toxin
☒	☐	Any other potentially harmful combination of experiments and agents

Plants

Seed stocks	ddm1-1; rdr1 (SALK_112300); rdr2 (SALK_059661); rdr6-11; kyp-4 (SALK_044606); tailswap cenh3;
Novel plant genotypes	epi-recombinant lines were generated by crossing rdr1 rdr2 ddm1 to rdr1 rdr2 RDR6/rdr6, as described in Methods.<br>Hairpin-complemented lines were performed by Agrobacterium mediated transformation, as described in Methods.<br>rdr1 rdr2 rdr6 ddm1 suppressors were generated with EMS screens, as described in Methods.
Authentication	The lines generated were genotyped by whole genome sequencing, their phenotypes were assessed by whole genome DNA methylation analysis, immuno-fluorescence imaging and cytogenetics. Further details are described in Methods.

ChIP-seq

Data deposition

☒ Confirm that both raw and final processed data have been deposited in a public database such as GEO.

☒ Confirm that you have deposited or provided access to graph files (e.g. BED files) for the called peaks.

Data access links<br>*May remain private before publication.*	https://www.ncbi.nlm.nih.gov/geo/query/acc.cgi?acc=GSE132005
Files in database submission	H3K9me2 IP and corresponding inputs for rdr1;2;6, rdr1;2;ddm1, rdr1;2;6;ddm1 and rdr1;2;6;ddm1;Cen5-Athila5<br>CENH3 IP and corresponding inputs for WT, rdr1;2;6, rdr1;2;ddm1, rdr1;2;6;ddm1 and rdr1;2;6;ddm1;Cen5-Athila5
Genome browser session<br>(e.g. UCSC)	NA

Methodology

Replicates	2 Biological replicates per genotype
Sequencing depth	All samples were sequenced paired-end 76bp, generating at least 20M raw reads each with minimum 80% mapping efficiency.

Antibodies	anti-H3K9me2 (ab1220, Abcam); anti-CENH3 (gift from Prof. S. Henikoff)
Peak calling parameters	MACS2 with default parameters (q value = 0.01)
Data quality	Data quality was assessed by mapping statistics, correlation between replicates and browser visualization confirming previously reported patterns.
Software	ChIP-seq analysis is described in Methods, using bowtie2 to map, and MACS2 to call peaks
