# Supplementary figures and images for: Pseudouridine guides germline small RNA transport and epigenetic inheritance
Source: Nat Struct Mol Biol. 2024 Sep 6;32(2):277–86. doi: 10.1038/s41594-024-01392-6 (PMC11832342; doi:10.1038/s41594-024-01392-6)

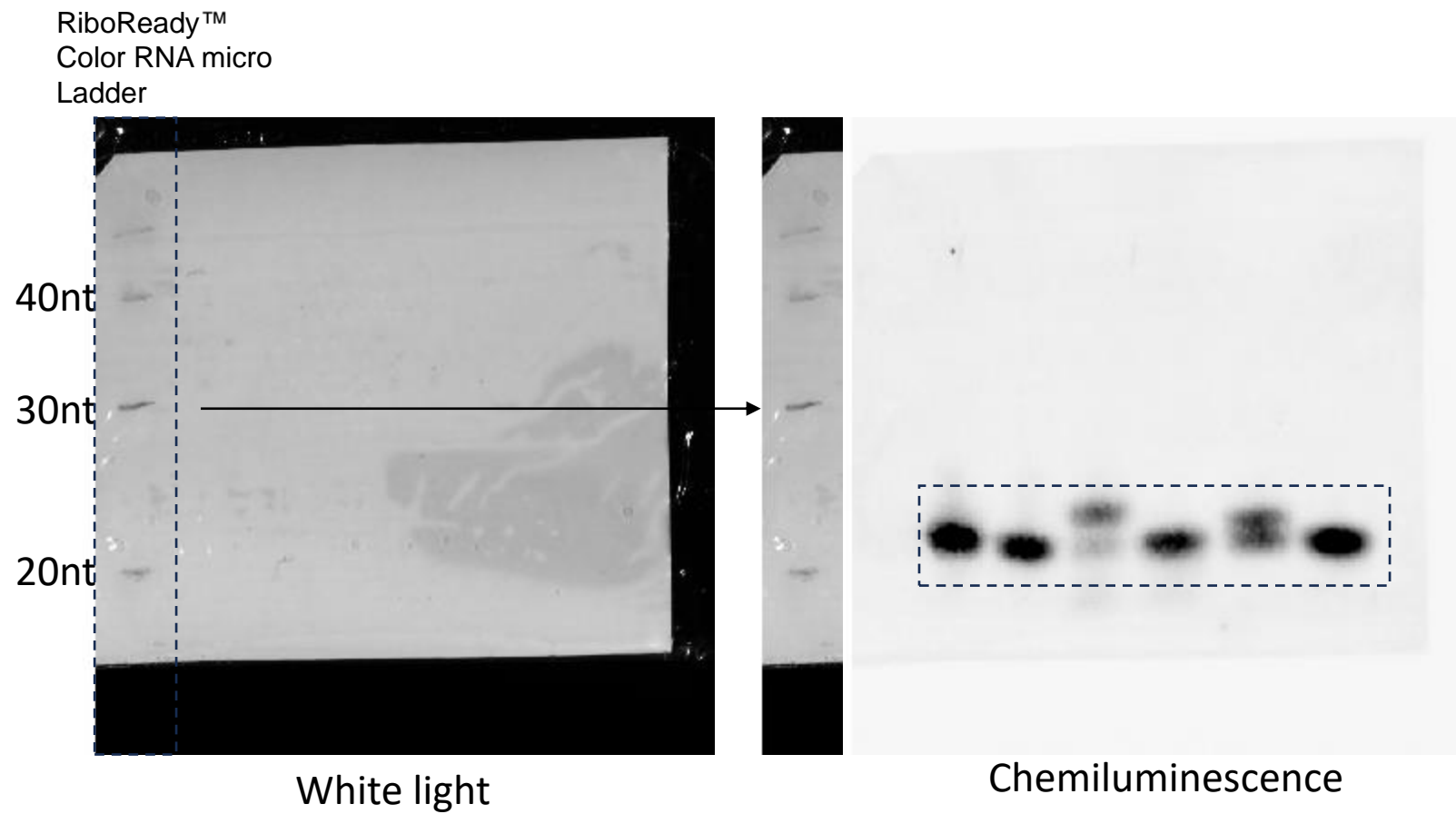

Figure 1h

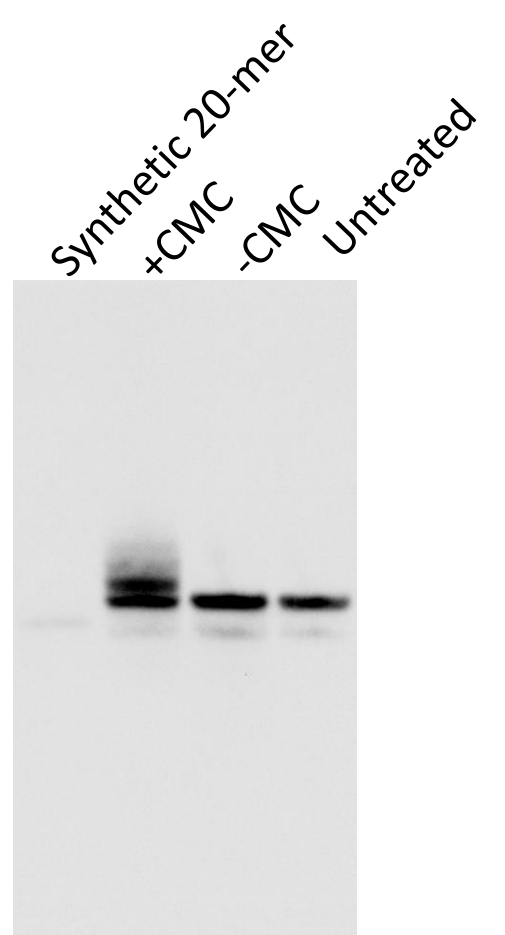

Figure 1i

Supplement: Supplementary file 3 — Unprocessed northern blots. [file 41594_2024_1392_MOESM3_ESM.pdf]
